# Hypertensive disorders in pregnancy complicated by liver rupture or hematoma: a systematic review of 391 reported cases

**DOI:** 10.1186/s13017-022-00444-w

**Published:** 2022-07-08

**Authors:** Goran Augustin, Matija Hadzic, Josip Juras, Slavko Oreskovic

**Affiliations:** 1 Department of Surgery, University Hospital Centre Zagreb, School of Medicine University of Zagreb, Kišpatićeva 12, 10000 Zagreb, Croatia; 2 School of Medicine, University of Zagreb, Šalata 2, 10000 Zagreb, Croatia; 3 Department of Surgery, Clinical Hospital ‘’Sveti Duh’’, Ul. Sveti Duh 64, 10000 Zagreb, Croatia; 4 Department of Obstetrics and Gynecology, University Hospital Centre Zagreb, School of Medicine, University of Zagreb, Petrova 13, 10000 Zagreb, Croatia

**Keywords:** Liver hematoma, Liver rupture, Pregnancy, Puerperium, Preeclampsia, Eclampsia, Cesarean delivery, Fetal death, HELLP syndrome, Treatment

## Abstract

**Background:**

Spontaneous liver rupture in pregnancy is often unrecognized, highly lethal, and not completely understood. The goal was to summarize and define the etiology, risk factors, clinical presentation, appropriate diagnostic methods, and therapeutic options for spontaneous hepatic rupture during pregnancy/puerperium (SHRP) complicated by the hypertensive disorder.

**Methods:**

Literature search of all full-text articles included PubMed (1946–2021), PubMed Central (1900–2021), and Google Scholar. Case reports of a spontaneous hepatic rupture or liver hematoma during pregnancy or puerperium as a complication of hypertensive disorders (preeclampsia, eclampsia, HELLP syndrome) were searched. There was no restriction of language to collect the cases. Additional cases were identified by reviewing references of retrieved studies. PRISMA guidelines for the data extraction and quality assessment were applied.

**Results:**

Three hundred and ninety-one cases were collected. The median maternal age was 31 (range 17–48) years; 36.6% were nulliparous. Most (83.4%) occurred in the third trimester. Maternal and fetal mortality was 22.1% and 37.2%, respectively. Maternal and fetal mortality was significantly higher 1) before the year 1990, 2) with maternal hemodynamic instability, and 3) eclampsia. The most important risk factors for SHRP were preeclampsia and HELLP syndrome. Most women had right lobe affected (70.9%), followed by both lobes in 22.1% and left lobe in 6.9%. The most common surgical procedure was liver packing. Liver transplantation was performed in 4.7% with 100% survival. Maternal mortality with liver embolization was 3.0%. Higher gestational age increases fetal survival.

**Conclusion:**

The diagnosis and treatment of SHRP are often delayed, leading to high maternal and fetal mortality. SHRP should be excluded in hemodynamically unstable patients with preeclampsia/eclampsia or HELLP syndrome and right upper abdominal pain. Liver embolization and liver transplantation contribute to maternal survival. Maternal and fetal mortality was significantly higher before the year 1990. Hemodynamic instability, preeclampsia, and eclampsia have a significant negative influence on maternal survival.

**Level of evidence:**

Level V

**Supplementary Information:**

The online version contains supplementary material available at 10.1186/s13017-022-00444-w.

## Introduction

A spontaneous hepatic rupture in pregnancy/puerperium (SHRP) is often an unrecognized, extremely rare life-threatening condition primarily associated with preeclampsia, eclampsia, or HELLP (hemolysis, elevated liver enzymes, and low platelet count) syndrome [1]. James Abercrombie described the first clinical case in 1844 [2]. A limited number of cases have been reported. However, some cases related to general perinatal mortality without further clarification may correspond to SHRP, indicating underreported incidence [3]. The exact pathophysiology is not entirely understood, and there is a wide variation of the severity of symptoms and signs. Due to the rarity, the diagnosis and treatment are delayed, leading to high maternal/fetal mortality.

There are no systematic reviews or meta-analyses of observational studies on the subject. Therefore, we investigated etiology, incidence, risk factors, clinical presentation, diagnostic and treatment approaches, maternal and fetal outcomes, and their interrelations about SHRP.

## Materials and methods

### Study design

Two researchers (GA and MH) conducted a systematic literature search of PubMed (1946-2020), PubMed Central (1900-2020), and Google Scholars. The search terms were: “preeclampsia hepatic/liver rupture“, “eclampsia hepatic/liver rupture“, “HELLP syndrome hepatic/liver rupture“, “hepatic/liver rupture pregnancy“, “hepatic/liver hematoma pregnancy“, “subcapsular hematoma preeclampsia/eclampsia/HELLP syndrome“. There was no language restriction. The references of included articles were searched for other articles reporting hepatic hemorrhage or rupture associated with hypertension in pregnancy during the study period.

### Criteria for study selection

Full-text case reports and case series dealing with SHRP were collected. The exclusion criteria were: (a) traumatic hepatic rupture in pregnancy and puerperium, (b) hepatic rupture due to liver neoplasm, (c) hepatic rupture due to other liver disorders that were not associated with hypertension in pregnancy, and (d) articles on the topic only numbering the patients lacking essential data.

### Assessment of risk of bias

We assessed the risk of bias using guidelines proposed by the Agency for Healthcare Research and Quality. The bias was assessed across studies and outcomes [4]. The study is exempt from ethics approval because we synthesized data from previously published studies.

### Data quality assessment

We applied the PRISMA guidelines for the data extraction and quality assessment [5]. The examiners assessed the studies' methodologies according to the tool for evaluating the methodological quality of case reports and case series described by Murad et al. [6].

### Data extraction and synthesis

**Fig. 1:**
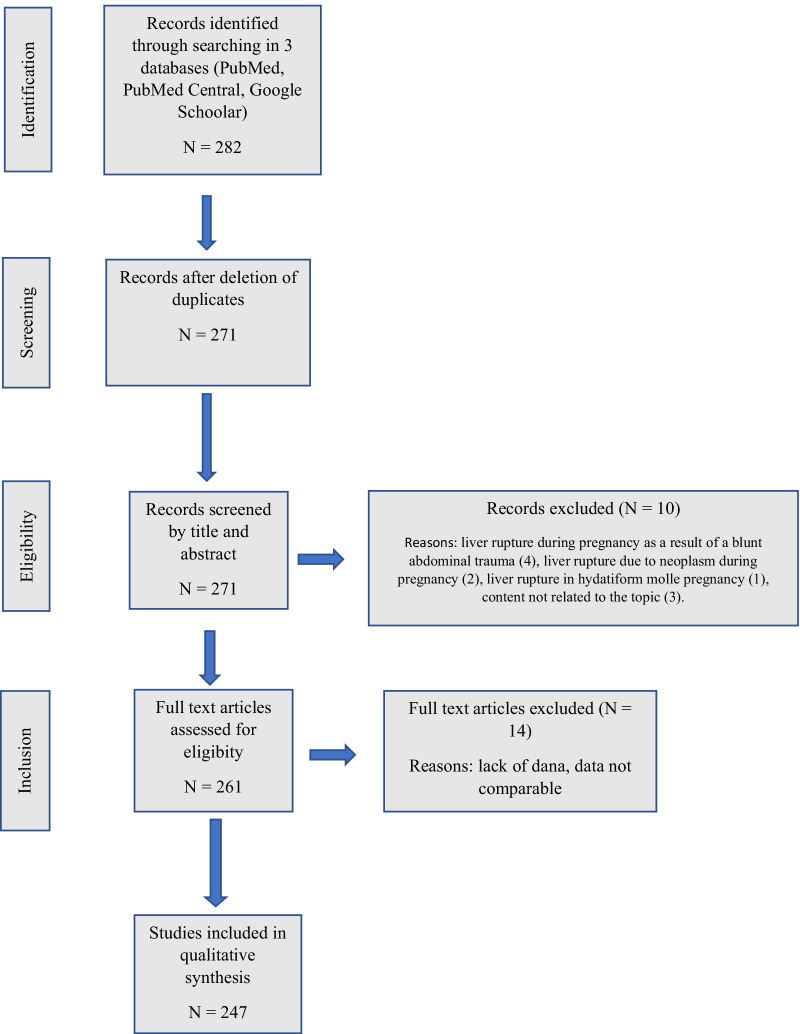
PRISMA search strategy flowchart

A total of 282 full-text articles was collected. The selection process followed the PRISMA workflow (Fig. 1). Ten articles were excluded for the following: liver rupture during pregnancy as a result of blunt abdominal trauma (n = 4), liver rupture due to neoplasm during pregnancy (n = 2), a liver rupture in a hydatidiform mole pregnancy (n = 1), content not related to the topic (n = 3). After removing duplicates (n = 11), an additional 14 cases were excluded due to incomparable data. Finally, 247 articles, including 391 cases, were included. Demographic data, hepatic rupture, hepatic hemorrhage without capsule rupture (subcapsular hematoma), hepatic lobe involvement, thrombocytopenia, class of HELLP syndrome, blood pressure, Cesarean section (CS), symptoms and signs, laboratory findings, diagnostic/treatment approaches, and maternal/fetal outcome were collected. Most outcome measures were unsuitable for meta-analysis, and we calculated crude estimates. Several interrelations were analyzed: (1) influence of maternal/gestational age and parity on the incidence of SHRP, (2) severity of preeclampsia/HELLP syndrome on the maternal/fetal outcome, (3) hepatic hemorrhage with or without capsule rupture on the outcome, (4) outcome related to the type of surgical treatment, and (5) outcome differences before the year 1990 and after.

All the investigators independently reviewed the collection forms to verify data accuracy.

### Statistical analysis

Categorical data are presented in absolute and relative frequencies. Numerical data are presented with the median and interquartile range (the Shapiro–Wilk test tested the normality of the distribution of numerical variables). The Kruskal–Wallis test tested the differences of numerical variables between more than two independent groups. The Mann–Whitney test was used for *post hoc* analysis, as it was used to explore the differences in independent variables between two groups. Differences in frequencies were tested by χ^2^ test and, if necessary, with Fisher exact test. The correlation of numerical variables was tested by the Spearman correlation coefficient rho (r_S_).

The further analysis explored outcome differences between the two periods—before and after 1990. The grounds for such division were increased use of new diagnostic and therapeutic procedures around 1990. We used binary logistic regression to explore the predictors of maternal mortality, considering the size of the standard error. All P values are two-sided, and the significance level was set to α = 0.05. The data were analyzed using MedCalc Statistical Software version 13.1.2 (MedCalc Software bvba, Ostend, Belgium; http://www.medcalc.org; 2014).

## Results

### General findings

A literature search revealed 391 cases of SHRP. Two hundred and fifty cases (63.9%) were diagnosed during pregnancy and 141 (36.1%) in the puerperium. The median age was 31 years (range 17–48), and the median gestational age was 35 weeks (interquartile range 31–37). Out of 347 women with available data, 127 (36.6%) were nulliparous, 168 (48.4%) had ≤ 4 deliveries, and 52 (15.0%) had > 4 deliveries.

**Fig. 2:**
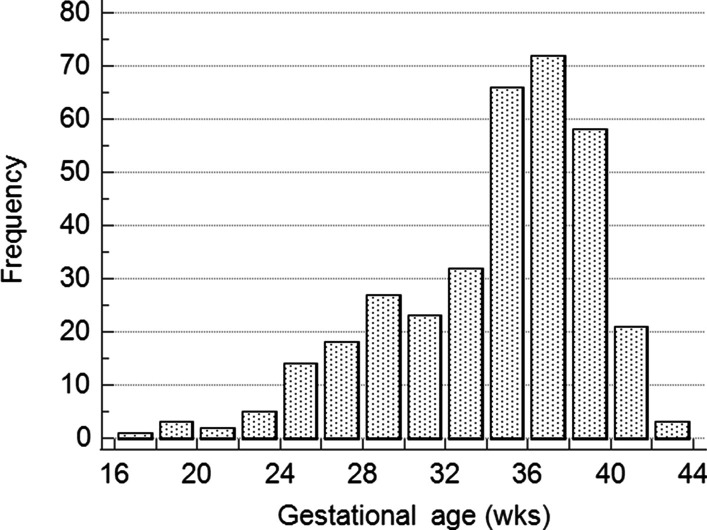
Distribution of spontaneous hepatic rupture during pregnancy and puerperium

Most cases of SHRP occurred during pregnancy (n = 250), mostly in the third trimester; of all cases, 114 (33.0%) occurred before 32 weeks, 114 (33.0%) from 32 to 36 weeks, and 117 (34.0%) from 37 weeks of gestation (Fig. 2). Trend analysis of SHRP (missing data for 15 patients) showed a significant difference in frequency being higher near or at term (χ^2^ = 261.678; *P* < 0.001). There was no significant difference between maternal mortality and the duration of gestation. Laboratory findings are listed in Table 1, but the exact time of blood sampling during the presentation could not be determined. The highest values were included for descriptive statistical analysis of preeclamptic patients with SHRP.

**Table 1:** Laboratory findings among patients with liver hematoma or liver rupture in relation to pregnancy/puerperium

	Pregnancy		Puerperium	
	Hematoma (n = 59)	Rupture (n = 191)	Hematoma (n = 48)	Rupture (n = 93)
Hemoglobin (g/L)	87.5 (69.0–104.0)	92.0 (71.0–112.0)	78.0 (65.0–88.0)	67.0 (60.0–86.0)
Platelets (×109)	67.85 (49.0–95.0)	65.5 (44.0–95.0)	61.0 (40.0–94.5)	55.0 (40.0–90.0)
Aspartate aminotransferase (U/L)	414.0 (117.0–1110.0)	547.0 (254.0–1011.0)	666.5 (350.5–2623.5)	791.5 (229.0–1560.0)
Alanine aminotransferase (U/L)	359.0 (146.0–1010.5)	500.0 (253.5–1057.0)	1146.0 (395.0–2737.0)	678.0 (246.0–1504.0)
LDH (U/L)	852.0 (567.0–1955.5)	978.0 (652.0–2011.0)	1728.0 (945.0–4180.0)	1420.0 (580.5–2910.0)
Systolic blood pressure (mmHg)	170 (148–188)	170 (150–190)	170 (150–180)	175 (160–190)
Diastolic blood pressure (mmHg)	100 (95–120)	106 (100–115)	100 (96–110)	100 (100–110)

Values presented as the median and interquartile range

A total of 86 women died (2 cases without data). Of all fetuses (n = 404), 146 died (12 cases without data), making fetal mortality 37.2%. More fetuses died among women with rupture (119/284 or 41.9%) than with hematoma (27/106 or 25.5% (χ^2^ = 8.841; *P* = 0.003)). CS was performed in 269 (72.1%) of 373 women (18 cases without data); 83 (22.2%) women had a vaginal delivery, 12 (3,2%) women died before delivery, 5 (1.3%) women had an abortion (16–21 week of gestation), 8 did not have data concerning the type of delivery (all fetuses died). There was no difference in the frequency of CS concerning the diagnosis of liver hematoma or rupture and the mother’s hemodynamic stability. Of 256 women with available data and CS, 75 (27.9%) had a liver hematoma and 194 (72.1%) had a liver rupture. Twenty-eight women (33.7%) with hematoma and 55 (66.3%) with rupture had a vaginal delivery, and no significant difference was found between the distribution of hematoma or rupture between women who had a vaginal delivery or CS (χ^2^ = 1.050; *P* = 0.305). There were 101 (39.0%) hemodynamically stable women with CS and 158 (61.0%) hemodynamically unstable women with CS. There was not difference compared to women with vaginal delivery, 32 (39.5%) hemodynamically stable and 49 (60.5%) hemodynamically unstable women (χ^2^ = 0.007; *P* = 0.935). Almost one-fourth of women (19/82 or 23.2%) died had a vaginal delivery, which was not significantly different compared to those who died and had CS (48/268 or 17.9%) (χ^2^ = 1.122; *P* = 0.289).

Two hundred and forty women had preeclampsia (50 cases without data). Eclampsia was recorded in 31 women (50 cases without data). HELLP syndrome was present in 267 women (63 cases without data). HELLP syndrome (Mississippi classification system) had significant differences in distribution; of 328 women with available data, 98 had Class 1, 123 Class 2, and 42 Class 3 HELLP syndrome (61 with no HELLP syndrome) (χ^2^ = 39.247; *P* < 0.001).

The frequencies of signs and symptoms related to pregnancy or puerperium are given in Table 2. For 48 (12.9%) women, symptoms and signs were not mentioned, 171 (46.1%) had at least one, 128 (34.5%) had two, and 24 (6.5%) had three signs or symptoms. Most women had right lobe affected (70.9%), followed by both lobes in 22.1%, and left lobe in 6.9%.

**Table 2:** The distribution of signs and symptoms in women with liver hematoma or rupture during pregnancy and puerperium*

Sign and symptoms	Antepartum	Postpartum	Total		
	N (%)	N (%)	N	*χ*^2^	*P*
Hypertension	172 (85.6)	102 (86.4)	274	0.046	0.830
Upper abdominal pain	204 (85.7)	105 (78.9)	309	2.807	0.094
Hemodynamic instability	148 (62.2)	84 (62.7)	232	0.009	0.924
Shoulder pain	30 (12.7)	19 (14.3)	49	0.196	0.658
Nausea/vomiting	66 (28.1)	24 (18.0)	90	4.634	0.031
Distended abdomen	11 (4.7)	40 (30.1)	51	45.384	< 0.001
Right lobe	127 (71.8)	78 (69.6)	205	0.161	0.689
Left lobe	16 (9.0)	4 (3.6)	20	3.100	0.078
Both lobes affected	34 (19.2)	30 (26.8)	64	2.290	0.130
Hematoma	59 (23.6)	48 (34.0)	107	4.946^†^	0.026
Rupture	191 (76.4)	93 (66.0)	284		
Preeclampsia	147 (67.7)	93 (75.0)	240	1.994	0.158
HELLP syndrome	170 (82.1)	97 (80.2)	267	0.194	0.660
Eclampsia	19 (9.1)	12 (9.8)	31	0.103	0.748

*Valid cases

^†^Test value for both, liver hematoma/rupture

### Hemodynamic instability

Data of deceased women show that 79/82 (96.3%) were hemodynamically unstable, significantly higher compared to stable women who died (χ^2^ = 52.324; *P* < 0.001). With hemodynamic instability, the odds ratio (OR) for maternal mortality was 23.89 (95% CI = 7.37–77.43, z = 5.290, *P* < 0.001). Pregnancy compared to puerperium does not influence maternal survival regarding hemodynamic instability (59/249 and 27/140, respectively; χ^2^ = 1.012; *P* = 0.315).

Women became hemodynamically unstable mostly before delivery, while about a quarter of women became hemodynamically unstable within 12 h from delivery. Of 235 women with data, 107 (45.5%) were unstable before delivery, 19 (8.1%) intrapartum, 56 (23.8%) during the first 12 h from delivery, 31 (13.2%) between 12 and 24 h, and 22 (9.6%) more than 24 hours from delivery. The analysis of patients who became hemodynamically unstable during delivery or within the first 24 hours after delivery showed that 40.0% of women who gave birth vaginally died, while maternal mortality after Cesarean section was 25.8% (χ2 = 2,181; *P* = 0.140). The difference in the percentage of maternal mortality is almost 15 %, and it would become statistically significant on a sample of 378 patients.

Among hemodynamically unstable women, 38 (36.5%) had a liver hematoma and 194 (72.4%) liver rupture (χ^2^ = 41.026; *P* < 0.001). There were 133/185 (71.9%) hemodynamically unstable women with the right lobe affected and 13 (7.0%) with the left liver lobe affected. Both lobes were affected in 39 (21.1%) hemodynamically unstable women. There was no significant difference in lobe involvement and hemodynamic instability (Pearson’s χ^2^ = 0.105; *P* = 0.949).

Fetal mortality was higher with maternal hemodynamic instability. Fetal mortality was 25.2% (35 cases) with hemodynamically stable women and 46.4% (108 cases) with hemodynamically unstable women (χ^2^ = 16.405; *P* < 0.001). Also, fetal mortality was higher with maternal liver rupture [117 cases (41.5%)] than liver hematoma [27 cases (25.2%)] (χ^2^ = 8.817; *P* = 0.003). Fetal death decreases with rising gestational age (r_S_ = −0.426; *P* < 0.001; n = 333).

### Preeclampsia, eclampsia, and HELLP syndrome

Preeclampsia carried a higher maternal mortality rate. Sixty women (25.1%) with preeclampsia died, while 13 (13.0%) without preeclampsia did not (χ^2^ = 6.114; *P* = 0.013). The same was found for eclampsia (48.4% vs. 18.2%, respectively) (χ^2^ = 15.520; *P* < 0.001). The ORs for maternal death with preeclampsia and eclampsia were 2.429 (95% CI 1.169–4.306; *P* = 0.015) and 3.705 (95% CI 1.970–9.035; *P < *0.001), respectively. Data on 86 women who died showed that 78 (90.7%) had a liver rupture and 8 (9.3%) had a liver hematoma. More women died with rupture (78/283) than with hematoma (8/106) (χ^2^ = 17.939; *P* < 0.001).

There were 267 (81.4%) women with HELLP syndrome (170 antenatally and 97 diagnosed in puerperium) and 61 without it. Among women who died, 18/61 (29.5%) did not have HELLP syndrome, and 46/265 (17.4%) who survived had it (χ^2^ = 4.639; *P* = 0.031). There was no difference between antenatal (29; 17.2%) and postnatal (17; 17.7%) mortality with HELLP syndrome (χ^2^ < 0.013; *P* = 0.910).

Of all 156 women with liver rupture during pregnancy, 130 (83.3%) had HELLP syndrome. Similar results were found when considering all women, including puerperium, [rupture with HELLP syndrome 195 (83.7%), without it 38 (16.3%)]. Rupture occurred in 46 (30.3%) women with the Class 1 HELLP syndrome, 60 (39.5%) Class 2, and 21 (13.8%) Class 3 HELLP syndrome (χ^2^ = 33.338; *P* < 0.001). No data exist for 48 patients.

### Diagnostic and operative procedures

Table 3 presents the frequencies of diagnostic and operative procedures. Laparoscopy was never used. Liver packing did not influence survival. Among women who survived 164/298 (55.0%) underwent liver packing, while 49/80 (61.3%) among women who died (χ^2^ = 0.991; *P* = 0.320). The same was found for hepatic artery ligation; 18/298 (6%) who survived had artery ligation, and 5/80 (6.3) who died (χ^2^ = 0.005; *P* = 0.944). Hepatorrhaphy and omentoplasty also did not influence survival; 39 (13.1%) women survived, and 14 (17.5%) died (χ^2^ = 1.019; *P* = 0.313). Liver resection did not influence maternal survival; 4/80 (5.0%) women with resection died and 7/298 (2.3%) survived (χ^2^ = 1.569; *P* = 0.210).

**Table 3:** Diagnostic and operative procedures with spontaneous liver rupture/hematoma during pregnancy*

	Antepartum	Postpartum	Total		
	N (%)	N (%)	N	χ^2^	P
*Diagnosis*					
Laparotomy	142 (58.7)	55 (39.3)	197	13.330	< 0.001
US	48 (19.8)	26 (18.6)	74	0.082	0.775
CT	20 (8.3)	32 (22.9)	52	15.997	< 0.001
US/CT	19 (7.6)	21 (15.0)	40	5.238	0.022
Autopsy	13 (5.4)	6 (4.3)	19	0.225	0.635
*Procedure*					
Laparotomy	194 (80.2)	89 (64.5)	283	11.355	0.001
Liver packing	143 (59.6)	70 (50.7)	213	2.795	0.095
Hepatic artery ligation	18 (7.5)	5 (3.6)	23	2.304	0.129
Liver embolization	18 (7.5)	15 (10.9)	33	1.277	0.259
Liver resection	8 (3.3)	3 (2.2)	11	0.417	0.519
Liver transplantation	10 (4.1)	4 (2.9)	14	0.386	0.534
Hepatorrhaphy/omentoplasty	38 (15.8)	15 (10.8)	53	1.860	0.173
*Maternal outcome*					
Survived	190 (76.3)	113 (80.7)	303	1.012†	0.315
Died	59 (23.7)	27 (19.3)	86		

^*^Valid cases; *US*—ultrasound, *CT*—computed tomography

^†^Test value for both, survived/died; χ^2^—Chi-square; *P*—statistical probability

Thirty-three women underwent embolization with one death (3.0%). Women who had embolization were less likely to die (OR 0.104; 95% CI 0.014–0.772; z = 2.212; *P* = 0.027). All women, 14/298 (4.7%), who underwent liver transplantation survived (χ^2^ = 3.951; *P* = 0.047).

A binary logistic regression explored the prediction of maternal mortality from preeclampsia/eclampsia, liver embolization, and hemodynamic instability. Of 391 cases, 75 were without data (the analysis was made for 297). The model accurately classified 81.3% of cases and was statistically significant; 27.5% of the variance of the maternal mortality could be explained by the model (Table 4). Preeclampsia/eclampsia and hemodynamic instability had a significant negative influence on maternal mortality.

**Table 4:** Predictive values of preeclampsia, eclampsia, liver embolization, and hemodynamic instability for maternal death

	B	SE	Wald	df	*P*	Exp(B)	95% CI for Exp(B)	
							Lower	Upper
Preeclampsia	0.749	0.381	3.856	1	0.050	2.115	1.001	4.467
Eclampsia	1.024	0.445	5.286	1	0.022	2.784	1.163	6.666
Liver embolisation	−1.839	1.056	3.032	1	0.082	0.159	0.020	1.260
Hemodynamic instability	2.721	0.610	19.907	1	< 0.001	15.193	4.598	50.202
Constant	−4.165	0.670	38.609	1	< 0.001	0.016		

R^2^ = 0.175 (Cox and Snell), 0.275 (Nagelkerke R^2^). Model χ^2^ (4) = 60.545; P < 0.001

*B*—regression coefficient, *SE*—Standard Error, *df*—degrees of freedom, *χ*^2^—chi-square; *P*—statistical probability, *CI*—Confidence Interval

### Comparison of data in two time periods

According to the changes in diagnostic and therapeutic approaches, analyzed data were divided into two periods. The first period included cases before the year 1990 (the “first period”) and the second after 1990 (the “second period”). The following results refer to the comparisons between these two periods.

There were 77 women with SHRP in the first period (28 postpartum) and 314 in the second period (113 postpartum). No difference existed for the frequency of liver rupture between the first (54/77; 70.1%) and the second period (230/314; 73.2%) (χ^2^ = 0.303; *P* = 0.582). No difference existed for age (the first period median age 31, range 17–44; the second period median age 31, range 18–48; Z = −1.672; *P* = 0.095), nor the distribution in age (categorically for every 5 years, χ^2^ = 2.815; *P* = 0.832) for women with SHRP. There were more primiparas in the second period (18.3% vs. 41.3%; χ^2^ = 17.200; *P* < 0.001).

There was no difference in the distribution of gestational weeks ( < 32 weeks, 32-36, 37-42, χ^2^ = 0.222; *P* = 0.895). Available laboratory findings and HELLP syndrome showed no statistically significant difference, except hemoglobin (Table 5). Systolic and diastolic blood pressure showed a difference, clinically insignificant. There were slightly more hypertensive and hemodynamically unstable women in the first period but more women with HELLP syndrome in the second period (Table 6). No difference in proportions was found for classes of HELLP syndrome (χ^2^ = 3.683; *P* = 0.159). The proportions of symptoms and signs did not show differences between the two periods. Data related to the proportion of signs and symptoms, preeclampsia, and its complications are shown in Table 6, along with the maternal outcomes. CS was more frequent in the second period [32/60 (53.3%) vs. 237/292 (81.2%); χ^2^ = 21.395; *P* < 0.001)].

**Table 5:** The comparison of laboratory parameters between two analyzed periods

Variable	Period (year)	n	C (25. to 75. P)	*Z*	*P*
Gestational age (weeks)	≤ 1990	56	35 (30–38)	−0.338	0.735
	> 1990	289	35 (31–37)		
Hb (g/L)	≤ 1990	23	92.0 (78.0–119.0)	−2.448	0.014
	> 1990	191	82.0 (62.0–100.0)		
Plt (×10^9^)	≤ 1990	17	50.0 (40.0–80.0)	−1.024	0.306
	> 1990	260	62.5 (44.5–80.0)		
AST (U/L)	≤ 1990	7	1011.0 (105.0–1235.0)	−0.315	0.753
	> 1990	235	562.0 (247.0–1266.0)		
ALT (U/L)	≤ 1990	8	560.5 (247.0–1470.0)	−0.307	0.759
	> 1990	215	530.0 (245.0–1286.0)		
LDH (U/L)	≤ 1990	2	1160 (304.0–2016.0)	−0.656	0.512
	> 1990	133	1021.0 (642.0–2220.0)		
Systolic blood pressure (mmHg)	≤ 1990	53	180 (160–214)	−3.025	0.002
	> 1990	186	170 (150–185)		
Diastolic blood pressure (mmHg)	≤ 1990	53	110 (100–126)	−4.307	< 0.001
	> 1990	186	100 (95–110)		

*C*—median; 25. *P.* *to 75*. P—interquartile range; *Z*—the value of Mann–Whitney test; *P*—statistical probability

**Table 6:** The comparison of symptoms and risk factors during two analyzed periods

Variable	Period (y)	N (%)	*χ*^2^	*P*
Upper abdominal pain	≤ 1990	63 (85.1)	0.226	0.634
	> 1990	246 (82.8)		
Shoulder pain	≤ 1990	13 (17.6)	1.505	0.220
	> 1990	36 (12.2)		
Nausea/vomiting	≤ 1990	16 (21.6)	0.403	0.526
	> 1990	74 (25.2)		
Distended abdomen	≤ 1990	11 (15.1)	0.098	0.754
	> 1990	40 (13.7)		
Right lobe involvement	≤ 1990	48 (72.7)	1.228*	0.541
	> 1990	157 (70.4)		
Left lobe involvement	≤ 1990	6 (9.1)		
	> 1990	14 (6.3)		
Both lobes involvement	≤ 1990	12 (18.2)		
	> 1990	52 (23.3)		
Liver hematoma/ rupture	≤ 1990	54 (70.1)	0.303	0.582
	> 1990	230 (73.2)		
Hypertension	≤ 1990	57 (95.0)	5.058	0.025
	> 1990	217 (83.8)		
Preeclampsia	≤ 1990	58 (92.1)	17.428	< 0.001
	> 1990	182 (65.5)		
Eclampsia	≤ 1990	7 (11.1)	0.382	0.537
	> 1990	24 (8.6)		
HELLP syndrome	≤ 1990	15 (31.9)	88.749	< 0.001
	> 1990	252 (89.7)		
Hemodynamic instability	≤ 1990	55 (74.3)	5.629	0.018
	> 1990	177 (59.4)		
Maternal death	≤ 1990	29 (38.2)	14.129	< 0.001
	> 1990	57 (18.2)		

*χ*^2^—Chi-square; *P*—statistical probability; *y*—year

*All lobes involvement (Pearson’s chi-square)

The first period had higher maternal and fetal mortality. Fetal mortality was recorded in 43 (56.6%) cases in the first period and 103 (33.7%) in the second period (χ^2^ = 13.481; *P* < 0.001). Maternal mortality was also significantly higher in the first period [26 (36.1%) vs. 56 (18.8%), (χ^2^ = 10.083; *P* = 0.001)]. Eclampsia was present in 4/21 (19.0%) women who died in the first period and 10/46 (21.7%) in the second period (χ^2^ = 0.063; *P* = 0.802). Similar results were observed for hemodynamic instability. In the first period, 28/29 (96.6%) who died were hemodynamically unstable, and 51/53 (96.2%) died in the second period (χ^2^0.008; *P* = 0.927).

There were differences in the definitive diagnostic modalities between these two periods (Table 7). In the first period, the diagnosis was based on laparotomy. In the second period, laparotomy and imaging techniques were equally diagnostic, especially comparing proportions of all imagining techniques (χ^2^ = 17.654; *P* < 0.001).

**Table 7:** Diagnostic and surgical techniques for liver hematoma/rupture compared for two periods

Variable	Period (y)	N (%)	*χ*^2^	*P*
*Diagnostic techniques*				
Laparotomy	≤ 1990	46 (62.2)	4.152	0.042
	> 1990	151 (49.0)		
US	≤ 1990	4 (5.4)	11.413	0.001
	> 1990	70 (22.7)		
CT	≤ 1990	11 (14.9)	0.130	0.719
	> 1990	41 (13.3)		
US/CT	≤ 1990	1 (1.4)	8.078	0.005
	> 1990	39 (12.7)		
Autopsy	≤ 1990	12 (16.2)	24.239	< 0.001
	> 1990	7 (2.3)		
*Surgical techniques*				
Laparotomy	≤ 1990	54 (71.1)	0.585	0.444
	> 1990	229 (75.3)		
Liver packing	≤ 1990	33 (44.0)	5.801	0.016
	> 1990	180 (59.4)		
Hepatic artery ligation	≤ 1990	3 (4.0)	0.712	0.399
	> 1990	20 (6.6)		
Liver embolization	≤ 1990	6 (8.0)	0.059	0.808
	> 1990	27 (8.9)		
Liver resection	≤ 1990	5 (6.7)	4.673	0.031
	> 1990	6 (2.0)		
Hepatorrhaphy/omentoplasty	≤ 1990	17 (22.7)	5.860	0.015
	> 1990	36 (11.8)		
Liver transplantation	≤ 1990	0	3.586	0.058
	> 1990	14 (4.6)		

*US*—ultrasound; *CT*—computed tomography; *χ*^2^—chi-square; *P*—statistical probability; *y*—year

Table 7 shows therapeutic approaches during two periods. No difference in the proportions of the laparotomies was found, but there were slight differences in the surgical techniques used. Liver resection, hepatorrhaphy, and omentoplasty were more common during the first period. All 14 women receiving liver transplantation were from the second period with 100% maternal survival.

## Discussion

SHRP is an exceedingly rare complication of pregnancy. Probably it is underreported due to high maternal and fetal mortality. Preeclampsia and HELLP syndrome are common in young primigravid women, while multiparous and older preeclamptic women are at higher risk of SHRP [7]. The reported incidence of SHRP is between 1/45,000 and 1/225,000 overall deliveries, whereas the incidence of SHRP in patients with HELLP syndrome is 0.4–1.8 % [8, 9]. The exact pathophysiology of SHRP is not entirely understood. Liver histology shows periportal hemorrhage and intravascular fibrin deposition. This can lead to hepatic sinusoidal obstruction, intrahepatic vascular congestion, and hepatic ischemia/infarction. Intraparenchymal and subcapsular hemorrhages may result in capsular rupture [10]. Thrombocytopenia from HELLP syndrome further enhances (uncontrolled) liver hemorrhage enhancing the risk of coagulopathy [11], making a vicious cycle.

We did not compare our results with previous reviews because these had fewer cases collected. Darby et al., in 2013, analyzed the relation of classes of HELLP syndrome with hepatic rupture and included ‘’only’’ 87 cases [12]. Vigil-de Gracia and Ortega-Paz, from 2012, collected ‘’only’’ 163 cases [13], while we have collected 391. Also, they have concentrated on the relation of HELLP syndrome, SHRP and mortality in general. We tried to extract more specific interrelations. Two recent reviews collected cases from 2000. One had 93 [11], while the other had 35 cases [14]. All these reviews had no definitive conclusions.

In our review, almost all patients presented in the third trimester or early postpartum. Most women had preeclampsia, and most women with SHRP had HELLP syndrome. This interrelation of SHRP and HELLP syndrome could be inaccurate due to variable definitions used. More women with HELLP syndrome survived. One of the reasons may be the severity of diagnosis with immediate antenatal care leading to earlier recognition of complications. Association with preeclampsia/eclampsia or HELLP syndrome is very high, almost a prerequisite for SHRP. Higher percentages of preeclampsia/eclampsia were diagnosed before 1990, while after 1990, HELLP syndrome was almost universal. This is probably due to more accurate diagnostics of HELLP syndrome. HELLP Class 2 was most common with SHRP, although SHRP can occur in all classes of HELLP syndrome.

There is a wide variation of the clinical presentations and the severity of symptoms and signs of SHRP. Some present with very mild symptoms before the sudden and massive circulatory collapse. The upper abdominal pain was the most frequent (83.3%), followed by hemodynamic instability (62.4%), nausea/vomiting (24.5%), and shoulder pain (13.2%). Almost half (46.1%) of patients had at least one, 34.5% at least two, and 6.5% at least three symptoms or signs. However, 12.9% of women were asymptomatic. Therefore, a lack of specific symptoms or signs leads to diagnostic dilemmas and delays in definitive diagnosis and management.

Most hemodynamically unstable women (majority with liver rupture) or those who developed eclampsia died. Both conditions were the only predictors of mortality. Also, these conditions remain frequent in both analyzed periods. Over 96% of hemodynamically unstable women died in both periods. Hemodynamic instability is highly lethal despite modern diagnostics and treatment options. Less than 10% become hemodynamically unstable more than 24 hours from delivery. Hemodynamical instability was not associated with the difference in number or liver lobe involvement. Fetal mortality was associated with the same conditions, or to be more precise, it is associated with maternal mortality. Fetal survival rises with gestational age.

Most pregnancies were completed by CS, with a higher incidence in the second period (53.3% vs. 81.2%). The higher incidence of CS in the second period resulted from the earlier use and more accurate diagnostic modalities, while the fetus was still alive. Also, better interdisciplinary support, more primiparous women, advancements in obstetrics, intrapartum monitoring, and neonatal care during the last decades lead to successful CS and fetal outcomes. A relatively high rate of vaginal delivery results from intrapartum or postpartum SHRP. Increased intra-abdominal pressure during vaginal delivery, sometimes accompanied with Kristeller’s maneuver (forbidden after 2007), can result in SHRP.

Imaging modalities were more frequent during the second period. During the first period, the diagnosis was made mainly by laparotomy (62.2%), while the second period had an equal ratio of surgical and radiological diagnoses (ultrasound 22.7%, CT 13.3%, and combined 12.7%).

**Fig. 3:**
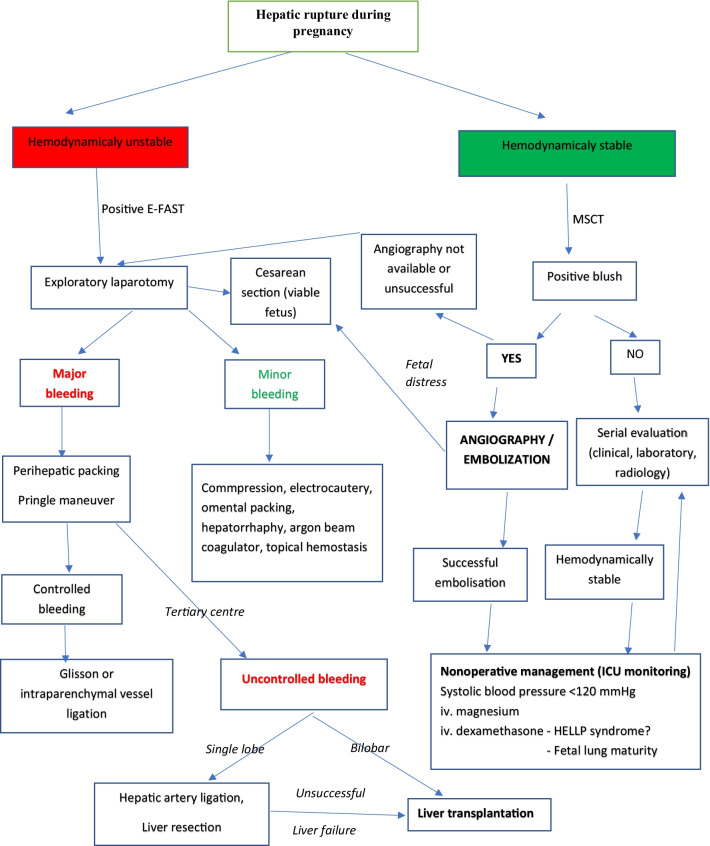
Therapeutic algorithm for spontaneous hepatic rupture in pregnancy

Maternal hemodynamic status dictates the diagnostic protocol. Extended focused abdominal sonography for trauma (E-FAST) immediately detects free intra-abdominal fluid. However, an abdominal ultrasound may be false-negative due to clotted blood or suboptimal quality views [15]. CT with iv. contrast is the gold standard in hemodynamically stable or stabilized patients (Fig. 3) [16]. Selective angiography can identify the bleeding site and stop the active hemorrhage [17, 18].

Preeclampsia was more common in the first period and HELLP syndrome in the second. Although HELLP syndrome is associated with higher mortality, due to more frequent use of diagnostic imaging, efficient modern treatment options, and advancements in intensive care, mortality was lower in the second period. This is confirmed by fewer hemodynamically unstable women in the second period.

Compared to the first period, maternal and fetal mortality after 1990 was 20% and 23.6% lower, respectively. Better survival could be partly explained by early imaging with correct diagnosis and adequate early intervention before the fetus died from maternal hemorrhagic shock.

The appropriate management varied from conservative with supportive therapy to surgical treatment with supportive therapy. Surgical options included liver packing, omental patch, hepatic artery ligation or percutaneous embolization, and liver transplantation. Liver packing was the most frequent, followed by hepatorrhaphy/omentoplasty, hepatic artery ligation, and liver resection. Laparoscopy was never used. However, those procedures did not influence maternal survival. Liver packing was sometimes combined with other procedures depending on the surgeon's preferences/skills, so it is difficult to evaluate the effectiveness of these treatment options and strategies for SHRP (Fig. 3).

Percutaneous hepatic artery embolization, a procedure increasingly used after 1990 [19], increases maternal survival, resulting in maternal mortality of 3.0%. The bias could be that stable patients underwent embolization while hemodynamically unstable were operated. Unfortunately, hepatic artery embolization was performed mainly in tertiary centers, so surgery was still the first option in many cases.

Factor VIIa was not analyzed in our review due to a small number of cases where its use was noted. It has been successfully used in several patients as an adjunct measure to main treatment for bleeding control [11], such as completed hepatic artery embolization with concomitant use of Factor VIIa [14]. Failure of conventional therapies and intolerability of massive blood transfusions are the circumstances where Factor VIIa may be considered although nonavailability, high cost, and risk of thromboembolism confine its use [11].

The only published article with an algorithm [20] is obsolete. In the meantime, many treatment options for SHRP during pregnancy were implemented, aside from surgical treatment. Guided by the WSES guidelines for liver trauma in the general population [16] and our results, we proposed an algorithm for SHRP in pregnancy (Fig. 1). Nonoperative management should be the initial treatment of hemodynamically stable patients with a subcapsular liver hematoma confirmed by a CT with iv. contrast. Monitoring in the intensive care unit with serial clinical examinations and laboratory testing is mandatory to detect further bleeding. Angiography with hepatic artery embolization may be a first-line intervention in hemodynamically stable patients with an arterial blush on CT. It results in a high bleeding control rate and high maternal survival.

Hemodynamically unstable and nonresponders to nonoperative management should undergo surgical exploration. The primary goal is the control of hemorrhage and bile leak and damage control resuscitation. Without major bleeding, digital compression with gauze, the use of electrocautery, bipolar devices, argon beam coagulation, topical hemostatic agents, simple suture of the hepatic parenchyma, or omental patching may stop the bleeding [16]. In cases of major hemorrhage, aggressive procedures include manual compression and hepatic packing, ligation of vessels, balloon tamponade, shunting procedures, or hepatic vascular isolation and exclusion [21]. Techniques vary significantly, especially between high- and low-income countries, leading to different mortality rates and trends in mortality. Major hepatic resections should be avoided during the initial operation. Resections should be considered in subsequent operations as a “resectional debridement” of large areas of devitalized liver done by a hepatobiliary surgeon.

’The final option for the successful treatment and maternal survival was liver transplantation. Most cases had a bilobar liver rupture or necrosis or complete right lobe rupture that extended to the left liver lobe, found at a series of laparotomies due to unsuccessful repeated liver packing. In the advanced stage, commonly only the left lateral liver segment appeared macroscopically normal. Most cases presented during or immediately after delivery. In one scenario, the most common surgical management included several laparotomies with unsuccessful liver packings with the addition of other unsuccessful hemostatic surgical techniques or liver resections. The other scenario included again one or more (un)successful liver packing, but the main indication for liver transplantation was not uncontrollable bleeding but irreversible or progressive liver failure. In both scenarios, the best strategy was to put a patient in Status One (high priority for the liver) of the liver transplant program in the early phase of the SHRP. In more fulminant cases, total hepatectomy was performed due to uncontrollable bleeding or liver failure with complete liver destruction along with multiorgan failure. The only option was to enroll a patient on the emergent liver transplant list as Status One priority and complete the operation with a temporary portacaval shunt until the receipt of the liver. In all cases, the anhepatic phase was less than 20 hours, and all patients survived.

Although the algorithm we propose is based on results, it is not binding, nor are the recommendations of many medical professional societies. The decision to terminate the pregnancy should be made in agreement with the informed patient, taking into account the ethical norms and rights of the patient.

### Strengths and limitations

The main strength of this study is the most extensive worldwide collection of SHRP during pregnancy. Limitations include nonstructured case descriptions and retrospective design. Some risk factors might be present but were not reported or measured. Therefore, the most important risk factor in our study, HELLP syndrome, could be falsely lower in the first period analyzed (before the year 1990), resulting in inaccurate comparison and conclusions. Accurate maternal and fetal survival rates are unmeasurable because many fatal cases are probably not published. The next issue is the accurate definition of the antepartum or postpartum SHRP. In some patients with preterm delivery, the diagnosis is made postpartum, but the rupture occurred in pregnancy or delivery, not puerperium although recognized in the puerperium. In this regard, the number of claimed cases during the puerperium could be objectively smaller. Also, SHRP detected during or after emergent CS is not always postpartum. Moreover, it is difficult to determine the exact times of the vital sign and laboratory measurements or imaging diagnostics for each case to make a particular conclusion.

In women with no symptoms reported, the question is whether these were hemodynamically unstable and therefore incapable of communicating and was the medical history adequate. Regarding the latter, there is a concern of whether these clinical presentations were objectively recorded. Nevertheless, the number of cases with missing data was not significant. Also, most patients had hypertensive disease as the cause of SHRP. Unfortunately, many of them had their last blood pressure measured before rupture. Therefore, the values do not correlate with blood pressure values that developed during rupture. Finally, the objective correlates of blood pressures and outcomes cannot be made. Also, there are no data about adjunct therapy for minimizing postoperative or postprocedural complications. For example, iv. magnesium can lower maternal blood pressure and also serve as a tocolytic. In HELLP syndrome management, dexamethasone is often used for fetal respiratory distress syndrome prophylaxis, but it is unclear whether it can reduce postoperative or postprocedural bleeding, minimizing surgical reinterventions.

### Conclusions and implications

This systematic review provided the most extensive findings on SHRP with the largest collection of patients. Hepatic involvement in patients with preeclampsia or HELLP syndrome is a life-threatening complication of pregnancy. Due to increased awareness, better diagnostic imaging, early fetal monitoring, and fetal and maternal supportive care, mortality has been reduced since the first described cases in the past century. Due to its rarity, most obstetricians are still not familiar with the SHRP. Patients with severe preeclampsia, eclampsia, or HELLP syndrome should be closely observed and treated to prevent such complications. Patients with hepatic involvement should be transferred to tertiary centers prepared for maternal/fetal complications. In cases with SHRP, a multidisciplinary approach provides the best outcome. Protocols should be determined for treating patients with hepatic involvement in pregnancy to prevent catastrophic complications.

## Supplementary Information

**Additional file 1** PRISMA checklist.

**Additional file 2** Table with all parameters from all included cases.

**Additional file 3**. References of all included cases.

## Data Availability

All data generated or analyzed during this study are included in this published article and its additional information files. The list of all references is included and uploaded as supplementary material.

## Abbreviations

SHRP: Spontaneous hepatic rupture during pregnancy/puerperium
HELLP syndrome: Hemolysis, Elevated Liver enzymes, and Low Platelet count
CS: Cesarean section
E-FAST: Extended-focused abdominal sonography for trauma
CT: computerized tomography
