# Supplementary material for: Hypertensive disorders in pregnancy complicated by liver rupture or hematoma: a systematic review of 391 reported cases
Source: World J Emerg Surg. 2022 Jul 8;17:40. doi: 10.1186/s13017-022-00444-w (PMC9270816; doi:10.1186/s13017-022-00444-w)
Supplement: Supplementary file 2 — Additional file 2 Table with all parameters from all included cases. [file 13017_2022_444_MOESM2_ESM.docx]

1. Abdi S, Cameron IC, Nakielny RA, Majeed AW. Spontaneous hepatic rupture and maternal death following an uncomplicated pregnancy and delivery. BJOG. 2001 Apr;108(4):431-3.
2. Abida A., Meddah J, El Youssfi M, and Bargach S. Hématome sous capsulaire du foie: A propos d’un cas. International Journal of Innovation and Applied Studies ISSN 2028-9324 Vol. 23 No. 4 Jul. 2018, pp. 700-704.
3. Aghav PA. Rupture of Subcapsular Hematoma of Liver in Severe Preeclampsia with HELLP syndrome: A Nightmare to an Obstetrician. Journal of Case Reports 2016;6(1):26-29
4. Alassia M, Buteler MM, Caffaratti C, Bollatti H, Caratti MM . HEMATOMA HEPÁTICO EN EL SÍNDROME HELLP, Revista de la Facultad de Ciencias Médicas 2011; 68(3):119
5. Aldemir M, Baç B, Taçyildiz I, Yağmur Y, Keleş C. Spontaneous liver hematoma and a hepatic rupture in HELLP syndrome: report of two cases. Surg Today. 2002;32(5):450-3.
6. Anyfantakis D, Kastanakis M, Fragiadakis G et al. Postpartum Spontaneous Subcapsular Hepatic Hematoma Related to Preeclampsia. Case Rep Emerg Med. 2014;2014:417406.
7. Araujo AC, Leao MD, Nobrega MH et al. Characteristics and treatment of hepatic rupture caused by HELLP syndrome. Am J Obstet Gynecol 2006; 195: 129–133.
8. Arias-Verdú MD, Prieto-Palomino MA, Balsera EC, Mora-Ordóñez J. Rotura espontánea del hígado en el síndrome HELLP. Med Clin (Barc).2006;126(19):757-9
9. Ayala-Haro N, Osorio Suarez CE, Alvarez-Jimenez V et al. Rotura hepática espontánea como complicación de síndrome de HELLP. Evid Med Invest Salud. 2016;9:134-6.
10. Aziz S, Merrell RC, Collins JA. Spontaneous hepatic hemorrhage during pregnancy. Am J Surg. 1983 Nov;146(5):680–682
11. Costa Barros B, Rezende MB: Hellp síndrome ocasionando ruptura hepática: Relato de Caso, Revista Ciências em Saúde 2019;9(1): 16-20 .
12. Batanero AS et al. HELLP SYNDROME COMPLICATED BY SUBCAPSULAR LIVER HEMATOMA. A SURGICAL EMERGENCY. HPB 2018;20 (2), 481.
13. Baumwol M, Park W. An acute abdomen : spontaneous rupture of liver during pregnancy. Br. J. Surg.(1976)Vol. 63; 718-720.
14. Benlbrikia K, Adrianjafy A., Kharbach A. and Baidada A. HEMATOME SOUS-CAPSULAIRE DU FOIE A 25 JOURS DU POST-PARTUM : A PROPOS D’UN CAS. nt. J. Adv. Res. 8(06), 1139-1142
15. Benetti Manfredini G et al. Síndrome de Hellp e rotura de hematoma hepático não precedido de preeclampsia. http://semanadoconhecimento.upf.br/download/anais-2014/biologicas/gabriela_benetti_manfredini-196029-relato_de_caso-sindrome.pdf
16. Berveiller P, Vandenbroucke L, Popowski T. Et al. Hepatic subcapsular hematoma: a case report and management update. J Gynecol Obstet Biol Reprod (Paris). 2012 Jun;41(4):378-82.
17. Bis KA, Waxman B. Rupture of the liver associated with pregnancy: a review of the literature and report of 2 cases. Obstet Gynecol Surv. 1976; 31:763–773
18. Boo Yang S. et al. Spontaneous Hepatic Rupture Associated with Preeclampsia: Treatment with Hepatic Artery Embolization. J Korean Soc Radiol 2010;63:29-32
19. Boormans EM, Bekedam DJ, Lenters E, Schoonderbeek FJ, Tilanus HW. Een spontane leverbloeding tijdens de zwangerschap: een zeldzame, levensbedreigende situatie. Ned Tijdschr Geneeskd. 2007 (26)1157-1162.
20. Borrás Suñer D, Fortuño Salais S, Díaz García C , Diago Almela V, Perales Marín A. Hepatic rupture associated with pre-eclampsia and HELLP syndrome, with catastrophic results. Prog Obstet Ginecol. 2009;52(7):402-6
21. Brito M, Gamito M, Neves AR, Caeiro F, Martins A, Dias E, Veríssimo C. Conservative management of a pregnancy complicated by preeclampsia and postpartum spontaneous hepatic rupture: A case report and review of the literature. Eur J Obstet Gynecol Reprod Biol. 2021 Dec;267:79-89. doi: 10.1016/j.ejogrb.2021.10.017. Epub 2021 Oct 18.
22. Browne CH, Hanson GC, de Jode LR, Roberts PA. Rupture of subcapsular haematoma of the liver in a case of eclampsia. Br J Surg. 1975 Mar;62(3):237-8.
23. Bruch H, Svroka L. Spontane Leberruptur nach Kaiserschnitteine seltene Komplikation der Eklampsie-Leber. Gcburtsh. u. Frauenheilk. (1982)42; 758-760
24. BURTON-BROWN, J R C, and J A SHEPHERD. “Rupture of the liver associated with parturition.” *British medical journal* vol. 1,4612 (1949): 941-3.
25. Calderon EG, Khawar S, Cunningham JA, Russell LD, Alpert MA. Pulmonary artery thrombus and subcapsular liver hematoma in a patient with HELLP syndrome: a therapeutic conundrum. Am J Med Sci. 2002;323(3):151-154.
26. Call M, Lorentzen D. Rupture of the liver in pregnancy- report of the case. Obstetrics and gynecology. 1965; Vol 25(4); 466-467.
27. Cappeller WA, Knitza R, Briegel J. et al. Subcapsular Hematoma of the Liver in HELLP Syndrome. An Interdisciplinary Emergency. Langenbecks Arch Chir 1992; 377 (2), 100-103.
28. Carazo H, Belén et al. Hematoma hepático subcapsular en el puerperio. Revista chilena de obstetricia y ginecología. 2013;78(6), 451-454.
29. Carlson KL, Bader CL. Ruptured subcapsular liver hematoma in pregnancy: a case report of nonsurgical management. Am J Obstet Gynecol. 2004 Feb;190(2):558-60.
30. Casasayas Argilagos G, Arañó PJF, Nápoles MD. Liver rupture associate with chronic hypertension and overadded preeclampsia. MediSan. 2018;22(09):1337-1246.
31. Cerda GP, Sánchez MM, García GR, et al. Síndrome de HELLP y ruptura hepática espontánea. Reporte de un caso y revisión de la literatura. Med Crit. 2003;17(4):138-143.
32. Cernea D, Dragoescu A, Novac M. HELLP Syndrome Complicated with Postpartum Subcapsular Ruptured Liver Hematoma and Purtscher-Like Retinopathy. Case Rep Obstet Gynecol. 2012;2012:856135.
33. Cerone DM, Catalino G. Spontaneous rupture of Liver during pregnancy. Obstetrics and gynecology. 12(4):459-461.
34. Cerwenka H, Bacher H, Werkgartner G, El-Shabrawi A, Mischinger H.J. Massive Liver Haemorrhage and Rupture Caused by HELLP-syndrome Treated by Collagen Fleeces Coated with Fibrin Glue. Eur J Surg 1998; 164: 709–711
35. Chan AD, Gerscovich EO. Imaging of Subcapsular Hepatic and Renal Hematomas in Pregnancy Complicated by Preeclampsia and the HELLP Syndrome. (1999);27;35-40.
36. Cheung H, Hamzah H. Liver rupture in pregnancy: a typical case? Singapore Medical Journal. 1992 Feb;33(1):89-91.
37. CH’MICHI N*, Benayada M, Bennani A. et al. SUBCAPSULAR LIVER HEMATOMA: 2 CASES REPORT AND LITTERATURE REVIEW. World Journal of Pharmacy and Pharmaceutical Sciences. 2020;9(10) 297-307.
38. Chou PY, Yu CH, Chen CC, Chen WT. Spontaneously ruptured subcapsular liver hematoma associated with hemolysis, elevated liver enzymes and low platelets (HELLP) syndrome. Taiwan J Obstet Gynecol. 2010 Jun;49(2):214-7.
39. Coelho T, Braga J, Sequeira M. Hepatic hematomas in pregnancy. Acta Obstet Gynecol Scand. 2000 Oct;79(10):884-6.
40. Colín-Cortes HM, Ruvalcaba-Carrillo R, Olivares-Revilla DM, et al. Hepatic subcapsular hematoma in HELLP syndrome. Report of 2 cases. Ginecol Obstet Mex. 2018;86(06):412-419.
41. Connor K, Rubin RA, Shrestha R, Johnson M, Sellers M, Butler B. ABO Incompatible Liver Transplantation as a Bridge to Treat HELLP Syndrome. Gastroenterology Research and Practice. 2009;2009:713937.
42. CRUZ-SANTIAGO, José et al. Hepatic rupture in HELLP syndrome: Surgical management guide. Cir. gen [online]. 2020, vol.42, n.1, pp.31-37. Epub 24-Sep-2021. ISSN 1405-0099. <https://doi.org/10.35366/92709>.
43. D’ambrosio R, Capasso L, Sgueglia S . Et al. Hepatic Haemorrhage in Pregnancy: A Case Report. G Chir. 2005 Jan-Feb;26(1-2):47-51.
44. Damiani GR, Confalonieri G, Fumagalli L et al. Severe Hepatic Rupture after Cesarean Section for Help Syndrome followed by Multiple Laparotomies: A Teaching Case. Gynecol Obstet. 2014;Vol 4, Issue 4 http://dx.doi.org/10.4172/2161-0932.1000218
45. Dart BW et al. A Novel Use of Recombinant Factor VIIa in HELLP Syndrome Associated with Spontaneous Hepatic Rupture and Abdominal Compartment Syndrome. J Trauma. (2004);57:171–174.
46. Das CJ, Srivastava DN, Debnath J. et al. Endovascular management of hepatic hemorrhage and subcapsular hematoma in HELLP syndrome. Indian J Gastroenterol. 2007 Sep-Oct;26(5):244-5.
47. DeHaan QC. Rupture of the liver associated with toxemia of pregnancy. Am J Obstet Gynecol. 1965 Sep 15;93:284-5.
48. DeKoninck PL, Loquet P, Leyman P et al. Spontaneous hepatic rupture in a normotensive monoamniotic twin pregnancy: case report and review of the literature. Gynecol Obstet Invest. 2010;70(1):69-72.
49. Descheemaeker PN, Compagnon P, Lavoué V. Et al. Liver transplantation for subcapsular haematoma during HELLP Syndrome. Ann Fr Anesth Reanim. 2009 Dec;28(12):1020-2.
50. Dessole S, Capobianco G, Virdis P, Rubattu G, Cosmi E, Porcu A. Hepatic rupture after caesarean section in a patient with HELLP syndrome:a case report and review of the literature. Arch Gynecol Obstet (2007) 276: 189-192.
51. Diksha Babu P, Tulsi B, Anuja T, Sushil K. Liver Rupture in Eclampsia: A Case Report. Global Journal for Research Analysis.2016;5(11); 253-254.
52. Dodson MG, O'Leary JA. Hepatic rupture during pregnancy. Obstet Gynecol. 1969 Jun;33(6):827-9.
53. Domingues AP , Gonçalves H, Costa J, Costa F, Maria Pais M, Paulo Moura P. Spontaneous rupture of a liver subcapsular hematoma during pregnancy. Hypertension in Pregnancy 25(supplement I):217 · January 2006 https://www.researchgate.net/publication/273133438_PP_Spontaneous_Rupture_Of_A_Liver_Hematoma_In_Pregnancy_-_A_Case_Report
54. Doumiri M, Elombila M, Oudghiri N, Saoud AT. Hématome sous-capsulaire du foie rompu compliquant une stéatose hépatique aiguë gravidique. The Pan African Medical Journal. 2014;19:38.
55. El Youssoufi S, Nsiri A, Salmi S, Miguil M. Liver rupture in peripartum: about 8 cases. J Gynecol Obstet Biol Reprod (Paris). 2007 Feb;36(1):57-61.
56. Elagwany AS et al. A fatal case of complicated HELLP syndrome and antepartum eclamptic fit with ruptured subcapsular liver hematoma.Apollo Medicine .2013 ;13 (4), 242 – 244.
57. Ellis BH, Skeoch T. Spontaneous rupture of the liver after delivery at term. Review of the literature with a case report. J Obstet Gynaecol Br Commonw. 1963 Dec;70:1060-2.
58. Erhard J, Lange R, Niebel W. et al. Acute liver necrosis in the HELLP syndrome: successful outcome after orthotopic liver transplantation. A case report. Transpl Int. 1993 May;6(3):179-81.
59. Escobar Vidarte MF, Montes D, Pérez A, Loaiza-Osorio S, Nieto Calvache A (2018): Hepatic rupture associated with preeclampsia,report of three cases and literature review. J Matern Fetal Neonatal Med. 2019;32(16):2767-2773.
60. Fat BC, Terzibachian JJ, Grisey A. Et al. Spontaneous hepatic rupture during an uncomplicated twin pregnancy. Gynecol Obstet Fertil. 2011 Jan;39(1):e7-e10.
61. Ferrer-Márquez M, Rico-Morales M, Belda-Lozano R, Yagüe-Martín E. Rotura hepática asociada a síndrome de HELLP. Cir Esp. 2008;83(3):155-6
62. Findeklee S. Fallbericht Leberruptur bei fulminantem HELLP-Syndrom in der 37. SSW [Case Report of Liver Rupture with Fulminant HELLP Syndrome in the 37th Gestational Week]. Z Geburtshilfe Neonatol. 2018;222(5):212-216.
63. Frise CJ, Davis P, Barker G, Wilkinson D, Mackillop L. Hepatic capsular rupture in pregnancy. Obstet Med. 2016 Dec;9(4):185-188.
64. Gadi N, V Sagar Pallepagu. Subcapsular liver rupture- A rare presentation in preeclampsia patient with congenital heart disease. Global Journal for research analysis 2019;8(2) https://www.doi.org/10.36106/gjra
65. [Garcarek](https://www.pubfacts.com/author/Jerzy+Garcarek) J,[Kurcz](https://www.pubfacts.com/author/Jacek+Kurcz) J,[Guziński](https://www.pubfacts.com/author/Maciej+Guzi%C5%84ski) M et al.Endovascular treatment of haemorrhagic complication of HELLP syndrome. Przegl Lek 2012 ;69(7):382-5
66. Ghorbanpour M, Makarchian HR, Yousefi B, Taghipour M. Conservative Management of Postpartum HELLP Syndrome and Intraparenchymal Liver Hematoma; A Case Report. Bull Emerg Trauma. 2019;7(2):196-198.
67. Gilboa Y, Bardin R, Feldberg D, Bachar GN. Postpartum hepatic rupture and retroperitoneal hematoma associated with HELLP syndrome. Isr Med Assoc J. 2006 Mar;8(3):219-20.
68. Golan A, White RG..Spontaneous rupture of the liver associated with pregnancy. A report of 5 cases. S Afr Med J. 1979 Jul 28;56(4):133-6.
69. Golmahammadlou S, Karjooyan T, Sane S et al. Spontaneous liver rupture in pregnancy complicating HELLP syndrome: case report. Tehran Univ Med J(TUMJ)2014 October;72(7):492-6.
70. Gonzales-Martinez G et al . Ruptura Hepática y Esplénica asociada con Preeclampsia severa: Presentación de un caso. Invest. Clín 2004; 45(1):63-68.
71. Gonzalez GD, Rubel HR, Giep NN, Bottsford JE Jr. Spontaneous hepatic rupture in pregnancy: management with hepatic artery ligation. South Med J. 1984 Feb;77(2):242-5. doi: 10.1097/00007611-198402000-00028. PMID: 6701595.
72. [Gordon SC](https://www.ncbi.nlm.nih.gov/pubmed/?term=Gordon%20SC%5BAuthor%5D&cauthor=true&cauthor_uid=1473680), [Meyer RA](https://www.ncbi.nlm.nih.gov/pubmed/?term=Meyer%20RA%5BAuthor%5D&cauthor=true&cauthor_uid=1473680), [Rosenberg BF](https://www.ncbi.nlm.nih.gov/pubmed/?term=Rosenberg%20BF%5BAuthor%5D&cauthor=true&cauthor_uid=1473680). Laparoscopic diagnosis of subcapsular hepatic hemorrhage in pre-eclamptic liver disease. Gastrointest Endosc. 1992 Nov-Dec;38(6):718-20.
73. Grand'Maison S, Sauvé N, Weber F. et al. Hepatic rupture in hemolysis, elevated liver enzymes, low platelets syndrome. Obstet Gynecol. 2012 Mar;119(3):617-25.
74. Greca FH, Coelho JC, Barros Filho OD, Wallbach A. Ultrasonographic diagnosis of spontaneous rupture of the liver in pregnancy. J Clin Ultrasound. 1984 Oct;12(8):515-6.
75. Greco Machado L, Sales do Amaral PH, Guimarães Silva M et al. Rotura hepática como complicação de pré-eclâmpsia grave: relato de caso. Rev Med Minas Gerais 2010; 20(4 Supl 2): 84-86.
76. Greenstein D, Henderson JM, Boyer TD. Liver Hemorrhage: Recurrent Episodes During Pregnancy Complicated by Preeclampsia. Gastroenterology( 1994);106:1666-1671.
77. Gupta, A., Joseph, S.R. and Jeffries, B. (2021), Managing a rare complication of HELLP syndrome in Australia: Spontaneous liver haematoma in pregnancy. Aust N Z J Obstet Gynaecol, 61: 188-194. <https://doi.org/10.1111/ajo.13318>
78. [Gutiérrez-Cafranga E](https://www.ncbi.nlm.nih.gov/pubmed/?term=Guti%C3%A9rrez-Cafranga%20E%5BAuthor%5D&cauthor=true&cauthor_uid=20617871) et al. Hepatic rupture and hemoperitoneum in a pregnant woman with HELLP syndrome. [Rev Esp Enferm Dig.](https://www.ncbi.nlm.nih.gov/pubmed/20617871) 2010 Jul;102(7):453-4.
79. Gutovich JM, Van Allan RJ. Hepatic Artery Embolization for Hepatic Rupture in HELLP Syndrome. J Vasc Interv Radiol. 2016 Dec;27(12):1931-1933.
80. Gyang AN, Srivastava G, Asaad K. Liver capsule rupture in eclampsia: treatment with hepatic artery embolisation. Arch Gynecol Obstet. 2006 Oct;274(6):377-9.
81. Habib et al: CONSERVATIVE APPROACH TO SUBCAPSULAR HEPATIC HEMATOMA SECONDARY TO POSTPARTUM HELLP SYNDROME, Critical Care Medicine: 2019;47(1):384.
82. Hackenberq R, Kußmman J, Schutz KD. Symptomatik eines rupturierten Leberhämatoms als schwere Komplikation des HELLP-Syndroms. Geburtsh. u. Frauenheilk. (1991)51; 313- 314.
83. Haidouri O, Benelbarhdadi I, Afifi R et al. Hématomes intrahépatiques causés par HELLP syndrome. J. Afr. Hépatol. Gastroentérol. (2012) 6:206-20.
84. HAKIM-ELAHI E. SPONTANEOUS RUPTURE OF THE LIVER IN PREGNANCY: REPORT OF A CASE AND REVIEW OF THE LITERATURE. Obstet Gynecol. 1965 Sep;26:435-40. PMID: 14341220
85. Haller AP, Abels DW, Straus R. Spontaneous rupture of the liver in a patient with nonconvulsive eclampsia. Am J Obstet Gynecol. 1951 Nov;62(5):1170-2.
86. Hamour OA, Hafiz AA. Post-partum spontaneous liver rupture: A case report. Saudi J Gastroenterol 1998;4:176-8
87. Han GH, Kim MA. Recurrent spontaneous hepatic rupture in pregnancy: A case report. Medicine (Baltimore). 2018;97(29):e11458. doi:10.1097/MD.0000000000011458
88. Harris BM, Kuczkowski KM.Diagnostic dilemma: hepatic rupture due to HELLP syndrome vs. Trauma. Arch Gynecol Obstet (2005) 272: 176–178.
89. Henríquez-Villaseca, María Paz, Catalán-Barahona, Alejandra, Lattus-Olmos, José, Vargas-Valdebenito, Karina, & Silva-Ruz, Solange. (2018). Hematoma subcapsular hepático roto en síndrome HELLP. Revista médica de Chile, 146(6), 753-761.
90. Hepburn IS. Pregnancy-Associated Liver Disorders. Dig Dis Sci (2008) 53:2346–2348
91. Herbert WN, Brenner WE. Improving survival with liver rupture complicating pregnancy. Am J Obstet Gynecol. 1982 Mar 1;142(5):530-4.
92. Hibbard LT. Spontaneous rupture of the liver in pregnancy: a report of eight cases. Am J Obstet Gynecol. 1976 Oct 1;126(3):334-8.
93. Hohlfeld P, Sanzeni W. Hematome sous-capsulaire du foie Arch Gynecol Obstet (1994) 255(Suppl 2): S255-S258.
94. Holst B, McGuinness E, Morris-Stiff G. Spontaneous peripartum liver haemorrhage presenting as foetal distress. Grand Rounds (2013)Vol 13; 42–46.
95. Hommann M, Schotte U, Richter K, K, Möller U, Basciani R, Scheele J: HELLP-Syndrom als Indikation für eine Lebertransplantation. Gynäkol Geburtshilfliche Rundsch 2001;(41) 8-11.
96. Horazeck C, Crockett CJ. Saved by the Massive Transfusion Protocol: A Case Report of an Obstetric Patient With Hemolysis, Elevated Liver Enzymes, and Low Platelet Count (HELLP) Syndrome and Glisson Capsule Rupture. A A Pract. 2019;12(11):409-411.
97. Howard PJ, Fandrichs TS. Spontaneous rupture of the liver in pregnancy; report of a case. Obstet Gynecol. 1956 Jan;7(1):40-3.
98. HuiKang Fan MM; Zhang, Ping PhD; Yang, Daji MM; Sun, Lu MM; Zhao, WenChao MM; Pan, Deng MM; Qi, Jun MD^∗^ HELLP syndrome complicated by subcapsular liver hematoma, Medicine Case Reports and Study Protocols: December 2020 - Volume 1 - Issue 2 - p e0020 doi: 10.1097/MD9.0000000000000020
99. Hummeida ME, Lewis M, Hussein I, Rayes DA. Pre-eclampsia, HELLP syndrome, abruptio placentae, postpartum haemorrhage, and postpartum rupture of subcapsular liver haematoma. NMJ June 2015 vol.3 No.16 ISSN 1858-6155
100. Hunter SK, Martin M, Benda JA, Zlatnik FJ. Livertransplant after massive spontaneous hepatic rupture inpregnancy complicated by preeclampsia. ObstetGynecol. 1995;85(5 Pt 2):819-22.
101. Ibrahim N, Payne E, Owen A.Spontaneous rupture of the liver in association with pregnancy. Case report. [Br J Obstet Gynaecol.](https://www.ncbi.nlm.nih.gov/pubmed/3994937) 1985 May;92(5):539-40.
102. Irvine LM. Massive non-obstetric postpartum haemorrhage. J Obstet Gynaecol. 2004 Feb;24(2):179-80.
103. Iwashita Y, Kan’o T, Hattori J. et al. A case of HELLP syndrome with multiple complications. Intern Med. 2012;51(16):2227-30.
104. Jimenez Rodrıguez RM, Pareja Ciuro F, Flores Cortes M, Sanchez ZV. Medical magement of the hepatic spontaneous rupture in pacient with HELLP syndrome. Med Clin (Barc). 2010;134(15):705–709
105. Juárez-Azpilcueta A, Motta-Martínez E, Montaño-Uzcanga A. Ruptura hepática como complicación de enfermedad hipertensiva del embarazo y síndrome de HELLP. Gac Méd Méx (2003)Vol.139 No. 3; 276-280.
106. Jungfleisch K, Fittschen M, Rapp HJ, Schäfer H, Bahlmann F. Liver rupture in HELLP syndrome. Perinat. Med. 2015; 4(1): 5–8
107. Kaltofen T, Grabmeier J, Weissenbacher T, Hallfeldt K, Mahner S, Hutter S. Liver rupture in a 28-year-old primigravida with superimposed pre-eclampsia and hemolysis, elevated liver enzyme levels, and low platelet count syndrome. J Obstet Gynaecol Res. 2019;45(5):1066-1070.
108. Kanonge TI, Chamunyonga F, Zakazaka N, Chidakwa C, Madziyire MG. Hepatic rupture from haematomas in patients with pre-eclampsia/eclampsia: a case series. Pan Afr Med J. 2018;31:86. Published 2018 Oct 4. doi:10.11604/pamj.2018.31.86.15975
109. Kapan M, Evsen MS, Gumus M, Onder A, Tekbas G. Subcapsular Liver Hematoma in HELLP Syndrome: Case Report. Gastroenterology Research 2010;3(3):144-146.
110. Karadia S, Walford C, McSwiney M, Nielsen MS. Hepatic rupture complicating eclampsia in pregnancy." British journal of anaesthesia 77.6 (1996): 792-794.
111. Karateke A, Silfeler D, Kurt R, Guler A, Kartal I. HELLP Syndrome Complicated by Subcapsular Hematoma of Liver: A Case Report and Review of the Literature. Case Rep Obstet Gynecol. 2014;2014:585672.
112. Kelly J, Ryan D, O’Brien N, Kirwan W. Second trimester hepatic rupture in a 35 year old nulliparous woman with HELLP syndrome: a case report. World Journal of Emergency Surgery : WJES. 2009;4:23.
113. King DR, de Moya MM, McKenney MG, Cohn SM. Modified Rapid Deployment Hemostat Terminates Bleeding From Hepatic Rupture in Third Trimester. J Trauma. 2006;61:739–742.
114. Kinthala S. et al. Subcapsular liver hematoma causing cardiac tamponade in HELLP syndrome. International Journal of Obstetric Anesthesia , Volume 21 (3). 276 – 279.
115. Knoop M, Neumann U, Neuhaus P. Bilobar liver hematoma and spontaneous rupture after cesarean section. Chirurg. 1999 Feb;70(2):214-6.
116. Kole M, Kole E, Kanbay S. A Rare Case Report: Subcapsular Hepatic Hematoma as a Complication of Preeclampsia. Asclepius Med Case Rep 2018;1(2):1-3.
117. Kramish D, Auer S, Reckler SM. Spontaneous rupture of the liver during pregnancy; review of the literature with case report. Obstet Gynecol. 1954 Jul;4(1):21-8.
118. Lavery DW, Bowes RM. Subcapsular haematoma of the liver in pregnancy: report on 4 cases. S Afr Med J. 1971 Jun 5;45(22):603-5.
119. Lee CS, Jung GY, Park CG. Et al. A Case of Subcapsular Hematoma of Liver During the Postpartum Period. Korean Society for Digestive Medicine. 1998;32:830 – 833.
120. [Lin TH](https://www.ncbi.nlm.nih.gov/pubmed/?term=Lin%20TH%5BAuthor%5D&cauthor=true&cauthor_uid=22515349), [Lien YR](https://www.ncbi.nlm.nih.gov/pubmed/?term=Lien%20YR%5BAuthor%5D&cauthor=true&cauthor_uid=22515349), [Lee CN](https://www.ncbi.nlm.nih.gov/pubmed/?term=Lee%20CN%5BAuthor%5D&cauthor=true&cauthor_uid=22515349). HELLP syndrome with postpartum hepatic infarction and subcapsular bleeding. [Acta Obstet Gynecol Scand.](https://www.ncbi.nlm.nih.gov/pubmed/22515349) 2012 May;91(5):637-8.
121. Links H. Spontaneous Liver Rupture Complicating Pregnancy. Br Med J 1946; 1 :275
122. Loevinger EH, Vujic I, Lee WM, Anderson MC. Hepatic rupture associated with pregnancy: treatment with transcatheter embolotherapy. Obstet Gynecol. 1985;65(2):281-284.
123. Loewenthal D. Spontane Leberruptur in der Schwangerschaft. Geburtsh a Frauenheilk. 1984(44); 819-820
124. López-Islas I, De la Cerda-Ángeles JC, Maxil-Sánchez AJ, et al. Hematomas hepáticos subcapsulares posparto en síndrome HELLP. Med Int Mex. 2019;35(2):302-307.
125. Mahi M, Chellaoui M, Nassar I et al. HÉMATOME SOUS-CAPSULAIRE DU FOIE ET GROSSESSE À PROPOS DE 4 OBSERVATIONS. J Radiol 2001;82:679-82.
126. Mallick IH, Syed SA, Kar AK. Liver rupture following delivery: HELLP needed. Emerg Med J. 2007 May;24(5):372.
127. Mamouni N, Derkaoui A, Bougern H, et al. Hématome sous capsulaire de foie compliquant une pré-éclampsie: à propos de 6 cas. The Pan Afr Med J. 2011;9:47.
128. Manas KJ, Welsh JD, Rankin RA, Miller DD. Hepatic hemorrhage without rupture in preeclampsia. N Engl J Med. 1985 Feb 14;312(7):424-6. doi: 10.1056/NEJM198502143120707. PMID: 3969097.
129. Mangieri Sobrinho F, Alves Pereira RM, Teruo Inoue I. Et al. Hepatic Rupture in Pregnancy – A Case Report. RBGO. 2002;24(2);129-132.
130. Marinaş MC, Mogoș G, Drăgușin RC, Tudorache Ş, Iliescu DG. Postpartum Spontaneous Subcapsular Hepatic Hematoma (SSHH)- Conservative Management. Case Report and Review of Literature. Curr Health Sci J. 2018;44(4):387-391.
131. Marsh FA, Kaufmann SJ, Bhabra K. Surviving hepatic rupture in pregnancy--a literature review with an illustrative case report, Journal of Obstetrics and Gynaecology, (2003) 23:2, 109-113.
132. Martín Martínez A, Sánchez Sánchez V, Bernaldo de Quirós I, García Hernández JA. Rotura hepática espontánea en el embarazo sin asociación con preeclampsia. Prog Obstet Ginecol 2003;46(4):181-4.
133. Martınez A ,et al. Rotura hepatica espontanea en el sındrome HELLP tratamiento mediante packing. Prog Obstet Ginecol. 2010;53(5):194—197
134. Martos Cano MA et al.Rotura hepatica espontanea en el embarazo.Prog Obstet Ginecol.2015. [http://dx.doi.org/10.1016/j.pog.2015.06.003[ahead](http://dx.doi.org/10.1016/j.pog.2015.06.003%5bahead) of print]
135. Mascarenhas R, Mathias J, Varadarajan R, Geoghegan J, Traynor O. Spontaneous hepatic rupture: a report of five cases. HPB : The Official Journal of the International Hepato Pancreato Biliary Association. 2002;4(4):167-170.
136. Matheï J, Janssen A, Olivier F, Depuydt P, Parmentier L, Harake R. Spontaneous Postpartum Subcapsular Liver Rupture. Acta chir belg, 2007(107) 713-715.
137. Matsuda Y, Maeda T, Hatae M. pontaneous rupture of the liver in an uncomplicated pregnancy. J Obstet Gynaecol Res. 1997 Oct;23(5):449-52.
138. Merchant SH, Mathew P, Vanderjagt TJ, Howdieshell TR, Crookston KP. Recombinant Factor VIIa in Management of Spontaneous Subcapsular Liver Hematoma Associated With Pregnancy. Obstet Gynecol. 2004 May;103(5 Pt 2):1055-8.
139. Messerschmidt L, Andersen LL, Sorensen MB. Postpartum HELLP syndrome and subcapsular liver haematoma. BMJ Case Rep 2014. doi:10.1136/bcr-2013-202503
140. Messina V, Dondossola D, Paleari MC, Fornoni G, Tubiolo D, Vergani P, Rona R, Rossi G. Liver Bleeding Due to HELLP Syndrome Treated With Embolization and Liver Transplantation: A Case Report and Review of the Literature. Front Surg. 2021 Nov 22;8:774702. doi: 10.3389/fsurg.2021.774702. PMID: 34881288; PMCID: PMC8646087.
141. Miguelote RF, Costa V, Vivas J et al. Postpartum spontaneous rupture of a liver hematoma associated with preeclampsia and HELLP syndrome. Arch Gynecol Obstet (2009) 279: 923-926.
142. Mikou MM, Sefrioui F, Harrandou M, Khatouf M, Kenjaa N. Surgical management of rupture of a subcapsular hepatic haematoma at 39 weeks' gestation. Ann Fr Anesth Reanim. 2008 Mar;27(3):271-2.
143. Millan CA, Forero JC. Right hepatectomy after spontaneous hepatic rupture in a patient with preeclampsia: A case report. Int J Surg Case Rep. 2017;39:250-252.
144. Mittal P, Dhattarwal SK, Sharma G ,Soni JP. SUDDEN MATERNAL DEATH DUE TO HELLP SYNDROME: AN AUTOPSY REPORT WITH CLINICAL AND PATHOLOGICAL REVIEW J Indian Acad Forensic Med. January - March 2017, Vol. 39, No. 1(107-110) DOI: 39. 10.5958/0974-0848.2017.00025.2
145. Mohamed MA, Salem MN, Salem AH, Hamady HR. Post-cesarean section rupture of subcapsular liver hematoma. A rare event should be kept in mind. Clinical and Experimental Obstetrics & Gynecology, 2018, 45(1): 141-142.
146. [Mokotoff R](https://www.ncbi.nlm.nih.gov/pubmed/?term=Mokotoff%20R%5BAuthor%5D&cauthor=true&cauthor_uid=5297854), [Weiss LS](https://www.ncbi.nlm.nih.gov/pubmed/?term=Weiss%20LS%5BAuthor%5D&cauthor=true&cauthor_uid=5297854), [Brandon LH](https://www.ncbi.nlm.nih.gov/pubmed/?term=Brandon%20LH%5BAuthor%5D&cauthor=true&cauthor_uid=5297854), [Carmillo MF](https://www.ncbi.nlm.nih.gov/pubmed/?term=Carmillo%20MF%5BAuthor%5D&cauthor=true&cauthor_uid=5297854). Liver rupture complicating toxemia of pregnancy. An example of thrombohemorrhagic disease. Arch Intern Med. 1967 Apr;119(4):375-80.
147. Moodley, N C Ngene: Spontaneous liver haematoma rupture associated withpre-eclampsia in a low- to middle-income country: Lessons to be learnt from maternal death assessments. S Afr Med J 2018;108(10):809-812.
148. Morgan GH, Gammill SL. Subcapsular hepatic hematoma without rupture, due to severe preeclampsia and the HELLP syndrome. J Tenn Med Assoc. 1987;80(12):736-737.
149. Moura C, Amaral L, Mendes J, et al. Hepatic rupture in HELLP syndrome. *J Surg Case Rep*. 2019;2019(10):rjz277. doi:10.1093/jscr/rjz277
150. Muñoz López C; Luco López V; Pérez, MV. Hematoma subcapsular hepático asociado a síndrome de HELLP en el puerperio / Hepatic subcapsular hematoma associated with HELLP syndrome in the puerperium. ARS med. 2018; 43(2): 52-56.
151. [Nagar](http://www.sciencedirect.com/science/article/pii/S0959289X04001001) MP,  [Gratrix](http://www.sciencedirect.com/science/article/pii/S0959289X04001001) AP, [O’Beirne](http://www.sciencedirect.com/science/article/pii/S0959289X04001001) HA,  [Enright](http://www.sciencedirect.com/science/article/pii/S0959289X04001001) SM. Survival following amniotic fluid embolism and cardiac arrest complicated by sub-capsular liver haematoma. International J of Obstetric Anesthesia.2005;14;1; 62–65.
152. Nam IC, Won JH, Kim S, Bae K, Jeon KN, Moon JI, Cho E, Park JE, Jang JY, Park SE. Transcatheter Arterial Embolization for Spontaneous Hepatic Rupture Associated with HELLP Syndrome: A Case Report. Medicina (Kaunas). 2021 Oct 2;57(10):1055. doi: 10.3390/medicina57101055.
153. Neerhof MG, Zelman W, Sullivan T. Hepatic rupture in pregnancy. Obstet Gynecol Surv. 1989 Jun;44(6):407-9. doi: 10.1097/00006254-198906000-00001. PMID: 2660034.
154. Nelson EW, Archibald L, Albo D Jr. Spontaneous hepatic rupture in pregnancy. Am J Surg. 1977 Dec;134(6):817-20.
155. Ng KB, Lim BH, Lees DA. Ruptured subcapsular haematoma of the liver in pregnancy; a case report. Eur J Obstet Gynecol Reprod Biol. 1990 Jul-Aug;36(1-2):179-83.
156. Ngene NC, Amin N, Moodley J. Ruptured subcapsular hematoma of the liver due to pre-eclampsia presenting as interstitial pregnancy and the role of intra-abdominal packing. Niger J Clin Pract 2015;18:300-3
157. Nguessan KL, Mian DB, Gondo D, Koffi A, Alla C. Ruptured subcapsular liver hematoma and pregnancy: a rare complication of severe preeclampsia: a report of a case discovered fortuitously at the Maternity Teaching Hospital of Cocody. Clin Exp Obstet Gynecol. 2012;39(4):550-2.
158. Niang MM, Aïdibé I, Cissé CT. Hématome sous-capsulaire du foie compliquant une toxémie gravidique. A propos d’une observation. Journal Africain de Chirurgie 2012;2(1):38-43.
159. [Nogales](http://www.sciencedirect.com/science/article/pii/S0210573X07745161) RM,  [Vázquez](http://www.sciencedirect.com/science/article/pii/S0210573X07745161) L,  [Pereira](http://www.sciencedirect.com/science/article/pii/S0210573X07745161) I. Et al. Hematoma subcapsular hepático, una complicación infrecuente de los estados hipertensivos del embarazo.Clínica e Investigación en Ginecología y Obstetricia. Dec (2007)Vol 34, Issue 6; 233-235.
160. Notelovitz M, Crichton D. Spontaneous rupture of the liver in pregnancy. S Afr Med J. 1968 May 11;42(19):476-80. PMID: 5657057
161. Nunes JO, Turner MA, Fulcher AS. Abdominal Imaging Features of HELLP Syndrome: A 10-Year Retrospective Review. AJR 2005; 185:1205–1210.
162. Ouvina Millan O , Alonso Vaquero MJ , Vidal Hernandez R, De Juan Barquin A. Hematoma subcapsular hepatico en el sındrome de HELLP. Prog Obstet Ginecol. 2010;53(9):373—376
163. Owen A, Kandalaft F. Spontaneous subcapsular haematoma and rupture of the liver during pregnancy. J Obstet Gynaecol Br Commonw. 1973 Sep;80(9):852-3.
164. Park JH, Lee YS, Oh YH et al. Spontaneous Hepatic Rupture in a Pregnant Woman withPreeclampsia: An Autopsy Case. Korean J Leg Med 2017;41:46-50.
165. Pavlis T, Aloizos S, Aravosita Pet al. Diagnosis and surgical management of spontaneous hepatic rupture associated with HELLP syndrome. J Surg Educ. 2009 May-Jun;66(3):163-7.
166. Peiro LZ, Salas R, Dolera Moreno C, Molla C. Rotura hepática espontánea en el síndrome HELLP. Med Intensiva. 2009;33(1):54-7
167. Peitsidou A, Peitsidis P, Contis J. Spontaneous Hepatic Rupture During Third Trimester of Pregnancy. Acta Chirurgica Belgica. (2008)108:4, 464-467.
168. Peñaflores-Rodríguez E, Téllez-Valdés JA, Barjau-Cadena R et al. Síndrome de HELLP y hematoma subcapsular hepático. Reporte de un caso. SALUD EN TABASCO 2007; 13(1): 597-601.
169. Perucca E, Domínguez C, González D et al. Rotura hepatica espontanea en sindrome de HELLP. Rev Chil Obstet Ginecol 2003; 68(1): 7-12 .
170. Pliego Pérez et al. Rotura hepatica espontánea durante el embarazo. Serie de cuatro casos y revision de la literature médica. Ginecol&Obst de Mex. 2006.Vol 74(4) 224-231.
171. Polo Gil M, Uriarte Rosquil E, Plaja Martí I, Del Río Manterola J, Lánderer Vázquez T, Remón Izquieta M. Hematoma hepático espontáneo en gestante a término durante el trabajo de parto [Spontaneous hepatic haematoma in a pregnant woman during labour]. An Sist Sanit Navar. 2017;40(2):295-297.
172. Portnuff J, Ballon S. Hepatic rupture in pregnancy.American Journal of Obstetrics & Gynecology , 1972;114(8) 1102 – 1104.
173. [Priddle HD](https://www.ncbi.nlm.nih.gov/pubmed/?term=PRIDDLE%20HD%5BAuthor%5D&cauthor=true&cauthor_uid=13016689), [Braden RG](https://www.ncbi.nlm.nih.gov/pubmed/?term=BRADEN%20RG%5BAuthor%5D&cauthor=true&cauthor_uid=13016689). Spontaneous hematoma of the liver in a woman with severe pre-eclampsia. Am J Obstet Gynecol. 1953 Jan;65(1):189-91.
174. Quesnel, A Weber, D Mendoza, D Garteiz. Hematoma hepático espontáneo en embarazo gemelar, Ginecol Obstet Mex 2012;80(2):110-114
175. Raga F, Sanz-Cort´e M, Bonilla-Musoles F. Three-dimensional ultrasound diagnosis of ruptured subcapsular liver hematoma caused by HELLP syndrome. Ultrasound Obstet Gynecol (2008); 32: 838–842
176. RAMIREZ CABRERA, Juan et al . Rotura hepática en síndrome de HELLP: electrofulguración y uso de malla. Reporte de caso. Rev. peru. ginecol. obstet., Lima , v. 65, n. 4, p. 537-540, Oct. 2019 . Available from <http://dev.scielo.org.pe/scielo.php?script=sci_arttext&pid=S2304-51322019000400019&lng=en&nrm=iso>. access on 20 Feb. 2022. http://dx.doi.org/10.31403/rpgo.v65i2222.
177. Reck T, Bussenius-Kammerer M, Ott R, Müller V, Beinder E, Hohenberger W. Surgical treatment of HELLP syndrome-associated liver rupture -- an update. Eur J Obstet Gynecol Reprod Biol. 2001 Nov;99(1):57-65.
178. Rademaker L. Spontaneous rupture of liver complicating pregnancy. Annals of Surgery. 1943;118(3):396-401.
179. Reséndiz Matamoros, Ruptura hepatica espontanea. Reporte de caso clinico y revision de la literatura <https://www.portalesmedicos.com/publicaciones/articles/3765/2/Ruptura-hepatica-espontanea.-Reporte-de-caso-clinico-y-revision-de-la-literatura>
180. Risseeuw JJ, de Vries JE, van Eyck J, Arabin B. Liver rupture postpartum associated with preeclampsia and HELLP syndrome. J Matern Fetal Med. 1999 Jan-Feb;8(1):32-5.
181. Rittenberry AB Jr, Arnold CL, Taslimi MM. Hemostatic wrapping of ruptured liver in two postpartum patients. Am J Obstet Gynecol. 1991 Sep;165(3):705-7.
182. Sahasranaman V, Daniel V, Fuentes G. Unusual Complication Of HELLP Syndrome – Hepatic Rupture. The Internet Journal of Emergency and Intensive Care Medicine. 2013 Volume 13 Number 1.
183. Sanabria-Padrón VH, Hernández-Valencia M, Castañeda-Valladares FE, Aceves-Solano JY. Tratamiento conservador del hematoma hepático subcapsular en pacientes con preeclampsia y síndrome de HELLP coexistentes: reporte de caso y revisión bibliográfica. Ginecol Obstet Mex (2013);81:414-419.
184. Sanchez-Bueno F, Garcıa Perez R, Torres Salmero G. Et al. Sindrome de HELLP con disfuncion hepatica severa: presentacio ´n de cuatro casos. Cir Esp . 2012;90(1) 33-37.
185. SANES S, KAMINSKI CA. Spontaneous rupture of the liver in eclampsia with fatal hemoperitoneum. Am J Obstet Gynecol. 1946 Aug;52:325-9. doi: 10.1016/s0002-9378(16)39844-1. PMID: 20993781.
186. Santos-Bolıvar J, Perozo-Romero J, Prieto-Montano J. et al. Ruptured subcapsular hepatic haematoma: a HELLP syndrome complication. Cir Esp. 2010;87(1):50-51.
187. Saporito WF et al. Ruptura Espôntanea de Fígado na Gestação Relato de Caso. Arq.med. ABC 1991;14(1): 36-38.
188. Saura P, Blanch L, Capdevila E. Et al. Spontaneous rupture of the liver during pregnancy. Intensive Care Med. 1995 Jan;21(1):95-6.
189. Schreinemakers C. Emergency Caesarean section complicated by preclampsia associated liver rupture. Anaesthesia Cases / 2013-0098/36-38.
190. Schwartz ML, Lien JM. Spontaneous liver hematoma in pregnancy not clearly associated with preeclampsia: a case presentation and literature review. Am J Obstet Gynecol. 1997 Jun;176(6):1328-32.
191. Sedlakova I, Podholova M, Tosner J. Subcapsular hepatic hematoma. International Journal of Gynecology and Obstetrics (2003)81; 299–300.
192. Seeler M, Behrend R, Morl FK, Körner J. Die spontane Leberruptur bei Eklampsie. Geburtsh. u. Fruucnhcilk . (1987)47; 346- 348
193. Segovia MR, Vázquez Mola GA. Liver hematoma associated with eclampsia and HELLP syndrome. Rev Nac (Itauguá) 2016;8(1):78-82.
194. Seren G, Morel J, Jospe R. Et al. HELLP syndrome and ruptured subcapsular hepatic haematoma. Case report and therapeutic options. Ann Fr Anesth Reanim. 2006 Oct;25(10):1067-9.
195. Shakya VC, Regmi MC, Sah P, Khaniya S, Adhikary S. An alarming but self-limited case of isolated large spontaneous liver hematoma in pregnancy. The Pan African Medical Journal. 2013;14:36.
196. Shames BD, Fernandez LA, Sollinger HW et al. Liver transplantation for HELLP syndrome. Liver Transpl.(2005) 11: 224–228.
197. Shaw C, Fattah N, Lynch D, Stokes M . Spontaneous Rupture of The Liver Following a Normal Pregnancy and Delivery Ir Med J. 2005 Jan; 98 (1): 27-28.
198. Shrivastava VK, Imagawa D, Wing DA. Argon Beam Coagulator for Treatment of Hepatic Rupture With Hemolysis, Elevated Liver Enzymes, Low Platelets (HELLP) Syndrome. Ostetrics & Gynecology Feb 2006 VOL. 107, NO. 2, PART 2, 525-526.
199. Siatti A, El Alaoui A,Baidada A et al. A successful conservative management of ruptured subcapsularhepatic hematoma with pre-eclampsia. PAMJ Clinical Medicine. 1. 10.11604/pamj-cm.2019.1.8.20755.
200. Sima Zué A, Bang Ntamack J, Biyogo P. Ruptured subcapsular haematoma of the liver. An observed case in an isolated maternity of Gabon. Ann Francaises d’Anesthesie et de Reanimation(2010);29 165–169.
201. Simic M, Tasic M, Stojiljkovic G, Draskovic D, Vukovic R. HELLP syndrome as a cause of unexpected rapid maternal death– A case report and review of the literature. Int J Legal Med (2005) 119: 103–106.
202. Singh Y, Kochar S, Biswas M, Singh K. Hepatic Rupture Complicating HELLP Syndrome in Pregnancy. Medical Journal, Armed Forces India. 2009;65(1):89-90.
203. Slattery LR, Abrams RM, Beranbaum ER, Labow SB, Aron B. Spontaneous hematoma of liver during pregnancy. Report of a case. Obstet Gynecol. 1968 Nov;32(5):664-9.
204. Smith LG Jr, Moise KJ Jr, Dildy GA 3rd, Carpenter RJ Jr. Spontaneous rupture of liver during pregnancy: current therapy. *Obstet Gynecol*. 1991;77(2):171-175.
205. Smyth BC. Spontaneous Hepatic Rupture Associated With HELLP Syndrome. Journal of Diagnostic Medical Sonography . 2011;27(1) 37 –39
206. [Sommer](http://www.ajronline.org/author/Sommer%2C+DG) DG, [Greenway](http://www.ajronline.org/author/Greenway%2C+GD) GD, [Bookstein](http://www.ajronline.org/author/Bookstein%2C+JJ) JJ,  [Orloff](http://www.ajronline.org/author/Orloff%2C+MJ) MJ. Hepatic rupture with toxemia of pregnancy: angiographic diagnosis. American Journal of Roentgenology (1979)132:3, 455-456.
207. Součková M, Pilka R, Malý T. Et al. HELLP syndrome complicated by liver rupture – case report. Čes. Gynek(2014);79; 219–225
208. Speert H, Tillman AJB. Spontaneous rupture of the liver in pregnancy, a rare complication of preeclampsia.AJOG.May 19; 63(5):1127-1132.
209. Srivastava G, Vine SJ, Asaad KA, Wolfe J. Successful outcome after hepatic rupture in previous eclamptic pregnancy. Arch Gynecol Obstet. 2007 Jul;276(1):73-5.
210. Stella CL, Malik KM, Sibai BM. HELLP syndrome: an atypical presentation. Am J Obstet Gynecol. 2008 May;198(5):e6-8.
211. Strate T, Broering DC, Bloechle C et al. Orthotopic liver transplantation for complicated HELLP Syndrome: Case report and review of the literature. Arch Gynecol Obstet (2000) 264:108–111.
212. Sujirachato K, Srisont S, Peonim V. HELLP syndrome in pregnancy as a cause of sudden unexpected death and spontaneous hepatic rupture: A medico-legal autopsy case report. J Med Assoc Thai. 2012 Apr;95(4):614-7.
213. Sutton BC, Dunn ST, Landrum J, Mielke G. Fatal postpartum spontaneous liver rupture: case report and literature review. J Forensic Sci 2008; 53: 472–475.
214. Tegene D, Regassa G, Usu A, Ayalew N. Successful Management of Intraoperatively Diagnosed Ruptured Spontaneous Sub-Capsular Liver Hematoma in a Woman with Preeclampsia. Int J Womens Health. 2021 Nov 30;13:1175-1179. doi: 10.2147/IJWH.S333905.
215. Teixeira de Freitas AC, Schulz G, Mori R, Coleho JC. HELLP syndrome and spontaneous liver rupture. ABCD Arq Bras Cir Dig. 2009;22(3):179-80.
216. Terasaki KH, Quinn MF, Lundell CJ, Finck EJ, Pentecost MJ. Spontaneous Hepatic Hemorrhage in Preeclampsia: Treatment with Hepatic Arterial Embolization. Radiology 1990; 174(3)1039-1041.
217. Thiessen, L, & Shaw, J. Hepatic rupture as the initial manifestation of HELLP syndrome. South African Journal of Surgery, 2018; 56(3), 30-31.
218. Troja A, Abdou A, Rapp C. et al. Management of Spontaneous Hepatic Rupture on Top of HELLP Syndrome: Case Report and Review of the Literature. Viszeralmedizin. 2015 Jun;31(3):205-8.
219. Turgut A, Özler A, Baflarano¤lu S, et al. A rare and catastrophic finding of HELLP syndrome: subcapsular hematoma and/or hepatic rupture. Perinatal Journal 2014;22(2):93-98
220. Tyagi V, Shamas AG, Cameron AD. Spontaneous subcapsular hematoma of liver in pregnancy of unknown etiology – Conservative management: A case report .The Journal of Maternal-Fetal and Neonatal Medicine, January 2010; 23(1): 107–110.
221. van de Minkelis JL, Steenvoorde P, Baranski AG. Liver rupture in a patient with HELLP syndrome successfully treated with extensive surgery combined with recombinant factor VIIa. Acta Chir Belg. 2006 Sep-Oct;106(5):602-4.
222. Vargas S, Teixeira E, Rodrigues G, Neves J, de Carvalh RM. Ruptured subcapsular liver hematoma during pregnancy: a lifesaving multidisciplinary approach. Acta Obstet Ginecol Port 2018;12(3):228-230.
223. Varotti G, Andorno E, Valente U. Liver transplantation for spontaneous hepatic rupture associated with HELLP syndrome. Int J Gynaecol Obstet. 2010 Oct;111(1):84-5.
224. Vera M. Eduardo, Pérez C. Alberto, Lattus O. Jose et al. Rotura hepatica asociada a preeclampsia severa y syndrome HELLP: Manejo y tratamiento con tamponamiento intraabdominal temporal de compresas. Rev Chil Obstet Ginecol 2004; 69(4):319-327.
225. Volz J, Volz E, Stoz F, Keckstein J. Spontanruptur der Leber bei HELLP-Syndrom. Geburtsh. u. Frauenheilk. (1992)52; 152- 156
226. Wagner WH, Lundell CJ, Donovan AJ. Percutaneous angiographic embolization for hepatic arterial hemorrhage. Arch Surg. 1985 Nov;120(11):1241-9. doi: 10.1001/archsurg.1985.01390350027007. PMID: 4051729.
227. Weemhoff A, Van Loon AJ, Aarinoudse JG. Ruptuur van de lever in de zwangerschap: een levensbedreigende complicatie bij het HELLP-syndroom. Ned Tijdschr Geneeskd (1996);I40(43) 2140-2142.
228. WEINGOLD AB, THOMPSON JW. Spontaneous rupture of the liver during pregnancy; case report and review of the literature. Am J Obstet Gynecol. 1960 Jul;80:155-60. doi: 10.1016/s0002-9378(16)36433-x. PMID: 13843593.
229. Westergaard L. Spontaneous rupture of the liver in pregnancy. Acta Obstet Gynecol Scand. 1980 (59):559-561.
230. Wicke C, Pereira PL, Neeser E, Flesch I, Rodegerdts EA, Becker HD. Subcapsular liver hematoma in HELLP syndrome: evaluation of diagnostic and therapeutic options‐a unicenter study. American Journal of Obstetrics and Gynecology. 2004;190(1):106‐112.
231. Wilson RH, Marshall BM.Postpartum rupture of a subcapsular hematoma of the liver. Acta Obstetricia et Gynecologica Scandinavica 1992;71: 394–397.
232. Wilson SG, White AD, Young AL, Davies MH, Pollard SG. The management of the surgical complications of HELLP syndrome. Ann R Coll Surg Engl 2014; 96: 512–516.
233. Woodhouse DR. Conservative management of spontaneous rupture of the liver in pregnancy. Case report. Br J Obstet Gynaecol. 1986 Oct;93(10):1097-9.
234. Wüst MD, Bolte AC, de Vries JI. Et al. Pregnancy outcome after previous pregnancy complicated by hepatic rupture. Hypertens Pregnancy. 2004;23(1):29-35.
235. Xavier P, Melo R, Amândio V, Beires J, Pereira-Leite L. Subcapsular hepatic hematoma in an otherwise uncomplicated pregnancy Arch Gynecol Obstet (2002) 266:44–45.
236. Xie Xiao-Xiao, Meng XF et al. Therapy of Liver Subcapsular Giant Hematoma with HELLP Syndrome: Case Report and Literature Review. Ann Clin Exp Hypertension 6(1): 1055 (2018).
237. [Yalcin](http://www.sciencedirect.com/science/article/pii/S1571467503001421) K, [Bilici](http://www.sciencedirect.com/science/article/pii/S1571467503001421) A, [Ayyildiz](http://www.sciencedirect.com/science/article/pii/S1571467503001421) O, [Degertekin](http://www.sciencedirect.com/science/article/pii/S1571467503001421) H, [Muftuoglu](http://www.sciencedirect.com/science/article/pii/S1571467503001421) E. Imaging of subcapsular liver hematoma and hepatic infarction in pregnancy complicated by the HELLP syndrome: a case report. European Journal of Radiology Extra 50 (2004) 21–26.
238. Yen SS. Spontaneous rupture of the liver during pregnancy. A report of two cases. Obstet Gynecol. 1964 May;23:783-7.
239. Yip RL, Pine DK. Spontaneous rupture of the liver: a rare complication of toxemia of pregnancy. Report of a case. Obstet Gynecol. 1966 Jul;28(1):70-2.
240. Yoshihara M, Mayama M, Ukai M. et al.Fulminant liver failure resulting from massive hepatic infarction associated with hemolysis, elevated liver enzymes, and low platelets syndrome. J. Obstet. Gynaecol. 2016 (42): 1375–1378
241. Yotsumoto G, Tanaka K, Ishizaki N, Komai A, Kawashima S, Taira A. Spontaneous Subcapsular Hepatic Hemorrhage Associated with Pregnancy: Report of a Case. Jpn J Surg (1997) 27:657-660.
242. You JS, Chung YE, Chung HS, Joo Y, Chung SP, Lee HS. Spontaneous hepatic rupture caused by hemolysis, elevated liver enzymes, and low platelet count syndrome. Am J Emerg Med. 2014 Jun;32(6):686.e3-4.
243. Zazula, R., Matějíčková, Š., Dutka, J., & Visokai, V. (2004). Poporodní spontánní ruptura jater při hellp syndromu. Interní Med. 2004; 6(10): 507-509
244. Zeirideen R, Kadir RA. Spontaneous postpartum hepatic rupture. Journal of Obstet&Gynaecol. 2009;Vol 29(2). 155-156.
245. Zhou X, Zhang M, Liu Z, Duan M, Dong L. A rare case of spontaneous hepatic rupture in a pregnant woman. BMC Pregnancy Childbirth. 2018;18(1):87. Published 2018 Apr 10. doi:10.1186/s12884-018-1713-5.
246. Zidouh S, Belyamani L, Kouach J, Drissi Kamili N. Subcapsular hepatic hematoma revealed by hemorrhagic shock in a preeclamptic patient. J Emerg Med. 2012 May;42(5):585-6.
247. Zissin R, Yaffe D, Fejgin M, Olsfanger D, Shapiro-Feinberg M. Hepatic infarction in preeclampsia as part of the HELLP syndrome: CT appearance. Abdom Imaging. 1999 Nov-Dec;24(6):594-6.
